# Esophageal cancer practice guidelines 2022 edited by the Japan esophageal society: part 1

**DOI:** 10.1007/s10388-023-00993-2

**Published:** 2023-03-18

**Authors:** Yuko Kitagawa, Ryu Ishihara, Hitoshi Ishikawa, Yoshinori Ito, Takashi Oyama, Tsuneo Oyama, Ken Kato, Hiroyuki Kato, Hirofumi Kawakubo, Hiroshi Kawachi, Shiko Kuribayashi, Koji Kono, Takashi Kojima, Hiroya Takeuchi, Takahiro Tsushima, Yasushi Toh, Kenji Nemoto, Eisuke Booka, Tomoki Makino, Satoru Matsuda, Hisahiro Matsubara, Masayuki Mano, Keiko Minashi, Tatsuya Miyazaki, Manabu Muto, Taiki Yamaji, Tomoki Yamatsuji, Masahiro Yoshida

**Affiliations:** 1 Department of Surgery, Keio University School of Medicine, 35 Shinanomachi, Shinjuku-Ku, Tokyo, 160-8582 Japan; 2 Department of Gastrointestinal Oncology, Osaka International Cancer Institute, Osaka, Japan; 3 QST Hospital, National Institutes for Quantum Science and Technology, Chiba, Japan; 4 Department of Radiation Oncology, Showa University School of Medicine, Tokyo, Japan; 5 Department of Hepato-Biliary-Pancreatic and Gastrointestinal Surgery, International University of Health and Welfare School of Medicine, Chiba, Japan; 6 Department of Endoscopy, Saku Central Hospital Advanced Care Center, Nagano, Japan; 7 Department Head and Neck, Esophageal Medical Oncology, National Cancer Center Hospital, Tokyo, Japan; 8 Kiryu Kosei General Hospital, Gunma, Japan; 9 Department of Pathology, Cancer Institute Hospital, Japanese Foundation for Cancer Research, Tokyo, Japan; 10 Department of Gastroenterology and Hepatology, Gunma University Graduate School of Medicine, Gunma, Japan; 11 Department of Gastrointestinal Tract Surgery, Fukushima Medical University, Fukushima, Japan; 12 Department of Gastroenterology and Gastrointestinal Oncology, National Cancer Center Hospital East, Chiba, Japan; 13 Department of Surgery, Hamamatsu University School of Medicine, Shizuoka, Japan; 14 Division of Gastrointestinal Oncology, Shizuoka Cancer Center, Shizuoka, Japan; 15 National Hospital Organization Kyushu Cancer Center, Fukuoka, Japan; 16 Department of Radiology, Yamagata University Graduate School of Medicine, Yamagata, Japan; 17 Department of Gastroenterological Surgery, Graduate School of Medicine, Osaka University, Osaka, Japan; 18 Department of Frontier Surgery, Graduate School of Medicine, Chiba University, Chiba, Japan; 19 Department of Central Laboratory and Surgical Pathology, National Hospital Organization Osaka National Hospital, Osaka, Japan; 20 Clinical Trial Promotion Department, Chiba Cancer Center, Chiba, Japan; 21 Department of Surgery, Japanese Red Cross Maebashi Hospital, Gunma, Japan; 22 Department of Clinical Oncology, Kyoto University Hospital, Kyoto, Japan; 23 Division of Epidemiology, National Cancer Center Institute for Cancer Control, Tokyo, Japan; 24 Department of General Surgery, Kawasaki Medical School, Okayama, Japan; 25 Department of Hepato-Biliary-Pancreatic and Gastrointestinal Surgery, School of Medicine, International University of Health and Welfare Ichikawa Hospital, Chiba, Japan

**Keywords:** Practice guidelines, Esophagus, Cancer

## Introduction

### Purpose of the guidelines

The primary objective of these guidelines was to provide general clinicians with information that would guide them to make informed choices from the available diagnosis/treatment strategies for esophageal cancer (intended only for malignant esophageal tumors of epithelial origin, and not for any other non-epithelial malignant tumors of the esophagus or metastatic malignant esophageal tumors). Furthermore, these guidelines are also intended as an aid for healthcare professionals other than physicians, and also for patients and their family members, to help them understand the fundamental principles of the diagnosis and treatment of esophageal cancer. These guidelines are intended to allow physicians to share the information contained therein and promote mutual understanding among healthcare professionals, patients, and the patients’ family members.

### Target users

The main target users of the guidelines are general clinicians and physicians specializing in the diagnosis and treatment of esophageal cancer. The guidelines also provide useful information to healthcare professionals other than physicians involved in the diagnosis and treatment of esophageal cancer, and also to the patients and their family members.

### Target patients

The guidelines are intended for adult patients with esophageal cancer and/or Barrett’s esophagus. While the mean age of patients diagnosed as having esophageal cancer is increasing with the ageing of the population, users should exercise caution when applying the guidelines to elderly patients aged 76 years or older, as many of the clinical studies from which evidence was drawn while formulating the guidelines involved patients who were 75 years or younger.

### Precautions for use

The guidelines are intended for standard diagnosis and treatment covered by the national health insurance system in Japan. Emphasis has been placed on evidence obtained from patients with esophageal squamous cell carcinoma, which is the most common histological type in East Asian countries, including Japan, while attention was also paid to the background and indications for treatment of esophageal carcinoma in Europe and the United States, where the main histological type is esophageal adenocarcinoma.

The guidelines are intended to guide physicians to provide standard diagnosis and treatment and not to force them to provide specific diagnosis and treatment. Since treatment of esophageal cancer is highly invasive and often requires treatment equipment (e.g., endoscopic treatment equipment, surgical treatment equipment, radiotherapy equipment, and intensive care unit) and human resources (multidisciplinary treatment team), individual diagnosis/treatment strategies should be determined according to the patient’s condition and the circumstances of the institution. Therefore, the person(s) directly in charge of diagnosis and treatment, and not the community or individuals involved in the development of the guidelines, will be responsible for the results of the diagnosis and treatment.

### Method of development of the esophageal cancer practice guideline

#### Scope formulation

The present revision of the guidelines was carried out based on the following:

1. Basic principles adopted for the preparation of the guidelines
  The basic principles for developing this 5th edition were deliberated upon at the meeting of the 1st Committee on Guidelines for Diagnosis and Treatment of Esophageal Cancer in April 2018. The present Edition continues to adopt the algorithm that provides a bird’s eye view of the entire flow of diagnosis and treatment of esophageal cancer and the detailed algorithms for each stage of the disease, which were newly introduced in the previous version of the guidelines. The committee agreed that Clinical Questions (CQs) related to debatable points in the algorithm that would require the physicians’ judgment in the clinical practice setting were to be extracted.
2. Major changes in the guidelines resulting from this revision
  Representatives of cooperative bodies including professionals other than physicians, and representatives of patients with esophageal cancer, were included as members of the Guideline Preparation Committee, and their multifaceted views were incorporated in the guidelines.
  A nationwide questionnaire survey was conducted, based on the results of which Quality Indicator (QI) studies would be conducted to determine the prevalence of usage of the guidelines and CQs would be created.
  In collaboration with the Japanese Gastric Cancer Association, the Japan Esophageal Society developed CQs relating to carcinoma of the esophagogastric junction and laid down the following ecommendations.
  A chemotherapy regimen for cStage IVB esophageal cancer recommended by the algorithm is shown.
  Systematic reviewers were transparently recruited.
3. On the methodology of preparation of the guidelines

The guidelines were prepared by referring to the Minds Manual for Guideline Development 2017 and 2020, issued by the Information Division of the Medical Information Network Distribution Service EBM (Minds), the Japan Council for Quality Health Care.

#### Preparation of CQs and search of the literature

The 41 CQs contained in the 4th edition of the guidelines were reexamined to exclude those related to treatments that had already become standard treatments from the 5th edition and are described in the text of the guidelines. New CQs, 39 in total, pertaining to clinically important problems, were prepared and included in the 5th edition of the guidelines. The Japan Medical Library Association was entrusted with a systematic search of the literature published on the treatment of esophageal carcinoma between January 2000 and August 2020, using keywords extracted from the CQs. The PubMed and Cochrane Library databases were used to search for articles in the English language, and the ICHUSHI-Web to search for articles published in Japanese.

Of the 39 CQs, 15 related to debatable points in the algorithm were adopted in the English version of the guidelines.

Moreover, a manual search was also conducted for articles/papers that had escaped retrieval by the systematic search and for those published after September 2020, as needed, based on information provided by the systematic review (SR) team and Guideline Preparation Committee members.

1. Inclusion criteria

Randomized controlled studies and observational researches among studies conducted in adult patients with esophageal cancer were adopted, in principle. Studies on accumulated cases, nevertheless, were also actively adopted, depending on the outcomes determined.

Only papers written in Japanese or English were adopted.

Contents of other documents, such as expert reviews and guidelines from other countries, were also reviewed in detail as reference data, although none of these was used as evidence.

(2) Exclusion criteria

Genetic studies and experimental studies in laboratory animals were excluded.

#### Systematic review procedure

For each of the CQs, the outcomes in terms of the balance between the benefits and risks were extracted and the level of importance thereof is presented. Each retrieved article was subjected to a primary and secondary screening, summarized, and then assessed for bias, besides classification of the study design. For each outcome in terms of the benefits and risks, individual papers were summed up and evaluated as a whole body of evidence, and the strength (certainty) of evidence was determined according to the Minds Manual for Guideline Development 2017 and 2020 (Table 1).

**Table 1:** Strength (certainty) of the whole body of evidence

A	Strong
	We have strong confidence that the estimated effect adequately supports the recommendation
B	Moderate
	We have moderate confidence that the estimated effect adequately supports the recommendation
C	Weak
	We have limited confidence that the estimated effect adequately supports the recommendation
D	Very weak
	We have very little confidence that the estimated effect adequately supports the recommendation

#### Determination of the strength of recommendations

The members of the Guideline Preparation Committee prepared drafts of the recommendation statements based on the results of a systematic review, and a consensus conference (online conference, because of the COVID-19 pandemic) was held to examine the strength of the recommendations. The strength of each recommendation was examined on the ground of the certainty of evidence, patient preferences, benefits and risks, and cost evaluation. As for the method of arriving at a consensus, a secret ballot was held with independent voting using a Google form, in accordance with the GRADE grid method; the strength of the recommendation was determined based on a ≥ 70% consensus. When a ≥ 70% consensus was not achieved in the first vote, a second vote was planned after the consultation; however, a ≥ 70% consensus was achieved in the first vote itself for all the CQs included in this 5th edition. When the Committee considered it difficult to determine the strength of recommendation for the CQ, a ballot was held in which “unable to determine the strength of the recommendation at present” was included in the choices, and a decision was made based on a ≥ 70% consensus.

The strength of recommendation was expressed in two directions × 2 steps as follows:

1. Conduct or non-conduct is “strongly recommended.”
2. Conduct or non-conduct is “weakly recommended.”

### Public hearing and external review

In May 2022, a draft of the guidelines was published on the website of the Japan Esophageal Society, inviting public comments from clinicians, other healthcare professionals, and patients.

The Guideline Preparation Committee examined the public comments, conducted a systematic review of important items, and revised the guidelines, as needed. In addition, The Guideline Review Committee independent from The Guideline Preparation Committee has been established to conduct external reviews.

### Revision

After publication of the guidelines, the Committee on Guidelines for Diagnosis and Treatment of Esophageal Cancer of the Japan Esophageal Society has taken the initiative to continue to review the contents of the guidelines and conduct public relations and dissemination/utilization activities. A revision of the guidelines is planned for approximately 5 years after publication of this guideline. In addition, a prompt report will be made when the results of a clinical study(ies) are published or in accordance with changes in the medical circumstances, such as revision of the health insurance coverage.

### Efforts related to public relations/dissemination (including plans)

#### Improvement of the method of guideline development

Improvement of flow charts, description of voter turnout, etc.

#### Improvement of convenience for users

Publication as a book and publication on the internet, free of charge (websites of the Japan Esophageal Society, Minds, Japan Society of Clinical Oncology, etc.), public lectures, public relations at meetings of scientific societies/study groups, etc.

### Conflict of Interest (COI) and Economic Independence

#### Conflict of interest (COI) reporting

Members of the Guideline Review Committee and Guideline Steering Committee personally reported their conflicts of interests in conformity with the regulations of the Japan Esophageal Society. The Board of Directors and the Ethics Committee of the Japan Esophageal Society confirmed the personally reported conflict-of-interest situations.

#### Restrictions at the recommendation decision conference based on COI

In case that any member who personally reports a COI (1) was an author of a paper that served as evidence for preparation of these guidelines (academic COI) or (2) has a COI concerning an enterprise or competing enterprise that manufactured and/or marketed a related drug(s) or medical device(s) (economical COI), the member will not be allowed to participate in the voting at the consensus conference, by self-declaration.

#### Efforts to prevent academic bias unique to the society

Efforts were made to avoid academic COI of any single academic organization, by constructing a cooperative system with a plurality of related academic bodies.

#### Economic independence

The Japan Esophageal Society met the entire expenditure for the preparation and publication of these guidelines and did not receive funding from any enterprises.

## Epidemiology, present status, and risk factors

### Summary

As for the dynamic trends of esophageal carcinoma in Japan, the incidence rate of this cancer has been constant or decreasing in men, while remaining constant or very gradually rising in women. The mortality rate has been decreasing in both men and women.

Among patients with this malignancy, the percentage of males is higher, as is the percentage of patients in their 60–70 s. The carcinoma is most frequently located in the middle thoracic esophagus, and squamous cell carcinoma is the overwhelmingly predominant histologic type. Esophageal cancer is known to be frequently associated with synchronous or metachronous multiple carcinomas.

The risk factors cited for esophageal squamous cell carcinoma include smoking and habitual alcohol consumption. On the other hand, a factor cited as protecting against development of this cancer is intake of vegetables and fruits. In regard to the risk factors for adenocarcinoma, Barrett’s epithelium arising from persistent inflammation of the lower esophagus due to gastroesophageal reflux disease (GERD) has been reported to serve as a risk factor for the development of esophageal carcinoma in Europe and the United States. In Japan, however, the risk of development of esophageal carcinoma associated with Barrett’s esophagus remains unclear because of the scarcity of documented cases.

### General remarks

#### Incidence and mortality

According to the statistics released by the Center for Cancer Control and Information Services, National Cancer Center, based on the cancer incidence (morbidity incidence rate) data derived from the Population-Based Cancer Registry, the estimated incidence rate of esophageal carcinoma (crude incidence rate) in 2015 was 31.2 persons per 100,000 population in men and 5.9 persons per 100,000 population in women. The age-adjusted incidence rate has been constant or decreasing in men recently, while remaining constant or very gradually increasing in women.

The vital statistics compiled by the Ministry of Health, Labour and Welfare showed that there were 11 619 deaths from esophageal carcinoma in 2019 (crude mortality rate: 9.4 persons per 100,000 population), accounting for 3.1% of all deaths from malignant neoplasms. The crude mortality rate associated with esophageal carcinoma in men was 15.9 persons per 100,000 population, ranking below the rates for cancers of the lung, stomach, colorectum, pancreas, liver and prostate, and the rate in women was 3.2 persons (per 100,000 population), ranking below the tenth place (http://ganjoho.jp/reg_stat/index.html). The age-adjusted mortality rate of esophageal carcinoma has been showing a downward trend in both men and women.

Cancer mortality data derived from vital statistics and from various graphs constructed based on those data are available at the Center for Cancer Control and Information Services, National Cancer Center (http://ganjoho.jp/reg_stat/index.html).

#### Present status of esophageal carcinoma in Japan

As for the present status of esophageal carcinoma in Japan, according to a nationwide survey conducted by the Japan Esophageal Society (8019 patients who were treated in 2013 and analyzed in 2019) [1], male patients outnumber female patients, with a male–female ratio of about 5.4:1, and most patients are in their 60 s or 70 s, these age groups accounting for about 70% of the patients overall. The carcinoma is predominantly located in the middle thoracic esophagus (in approximately 47% of cases), followed, in order of frequency, by the lower thoracic esophagus (in approximately 28% of cases), upper thoracic esophagus (in approximately 12% of cases), abdominal esophagus (in approximately 8% of cases), and cervical esophagus (in approximately 5% of cases). Squamous cell carcinoma is the overwhelmingly predominant histologic type, accounting for about 86% of all cases, followed in frequency by adenocarcinoma, including Barrett’s esophageal cancer, which accounts for about 7% of all cases. As for the treatment modalities, endoscopic treatment is performed in about 18% of cases, esophagectomy in about 61% of the cases, and chemotherapy/radiotherapy/chemoradiotherapy in about 51% of the cases.

### Risk factors

The most frequent risk factors for esophageal carcinoma identified in Japan are the smoking habit and habitual alcohol consumption. These are the most important risk factors for squamous cell carcinoma, being identified as risk factors in about 90% of all diagnosed cases of esophageal carcinoma in Japan. Concomitant use of tobacco and alcohol has been shown to be associated with a multiplied risk for the development of esophageal carcinoma [2–6]. In regard to the smoking habit in apparently healthy individuals, a systematic review was carried out to respond to “CQ1-1: Is smoking cessation recommended for healthy individuals from the perspective of preventing the development of esophageal carcinoma?”, and based on the systematic review, the recommendation statement was formulated and included in the previous edition of the guidelines: “There is strong evidence to recommend smoking cessation to apparently healthy individuals from the perspective of preventing the development of esophageal carcinoma”. No new evidence to contradict that recommendation has been reported since, and this CQ is not included in the present edition. In addition, for “CQ2: Is continued smoking and alcohol cessation recommended for patients cured of esophageal carcinoma?”, the following recommendation statement was formulated and included in the previous edition of the guidelines: “There is strong evidence to recommend continued smoking and alcohol cessation for patients cured of esophageal carcinoma”; therefore, this CQ was also excluded from the present edition of the guidelines.

As for habitual alcohol consumption, in October 2009, a working group of the World Health Organization specified that the acetaldehyde formed after consumption of alcoholic beverages is a Group 1 carcinogen [5]. In addition, genetic factors related to the capacity to metabolize alcohol or acetaldehyde were also reported to modify the risk of cancer associated with habitual alcohol consumption [7]. Since the strength of the recommendation regarding alcohol consumption for healthy individuals could not be established in the previous edition, CQ2 was formulated and included again in this edition to examine alcohol consumption in Asian heavy alcohol consumers.

In relation to dietary factors, poor nutritional status and vitamin deficiencies due to inadequate intake of fruits and vegetables have also been reported as risk factors for esophageal carcinoma. Since the intake of green and yellow vegetables and fruits has been suggested as a protective factor [8–10], a new CQ1 about the intake of vegetables and fruits was formulated.

Whilst adenocarcinoma accounts for only a small percentage of esophageal carcinoma patients in Japan, the percentage of esophageal carcinoma patients with this histological subtype is increasing in Europe and North America, and currently accounts for about more than half of all the cases of esophageal carcinoma in these regions. Barrett’s epithelium, caused by persistent inflammation of the lower esophagus due to GERD, is known as a predisposing lesion for the development of esophageal adenocarcinoma, and there are also reports of the contribution of GERD, high body mass index (BMI) (which is a risk factor for GERD), and smoking to the development of esophageal adenocarcinoma [11–14]. In Japan, however, no clear evidence has been established yet, because of the scarcity of cases of esophageal adenocarcinoma.

## Treatment algorithms for esophageal cancer and treatment policies based on the algorithm

### Japanese classification of esophageal cancer and tumor, node, metastasis (TNM) (Union for International Cancer Control [UICC]) classification

It should be noted that there exists some discordance on the subject of disease staging in this edition of the guidelines, as the disease staging was conducted in accordance with the Japanese Classification of Esophageal Cancer and the edition of the TNM (UICC) Classification prevailing at that time.

However, a by-histologic type classification system is adopted in the 8th Edition of TNM (UICC), in consideration of the difference in the prognosis between squamous cell carcinoma and adenocarcinoma, inferred largely from the therapeutic outcomes reported from Europe and the United States. In the present guidelines, the by-stage treatment algorithm is based on the 12th Edition of the Japan Esophageal Society’s Japanese Classification of Esophageal Cancer.

### Treatment algorithm for cStage 0 to I esophageal cancer (Fig. 1)

**Fig. 1:**
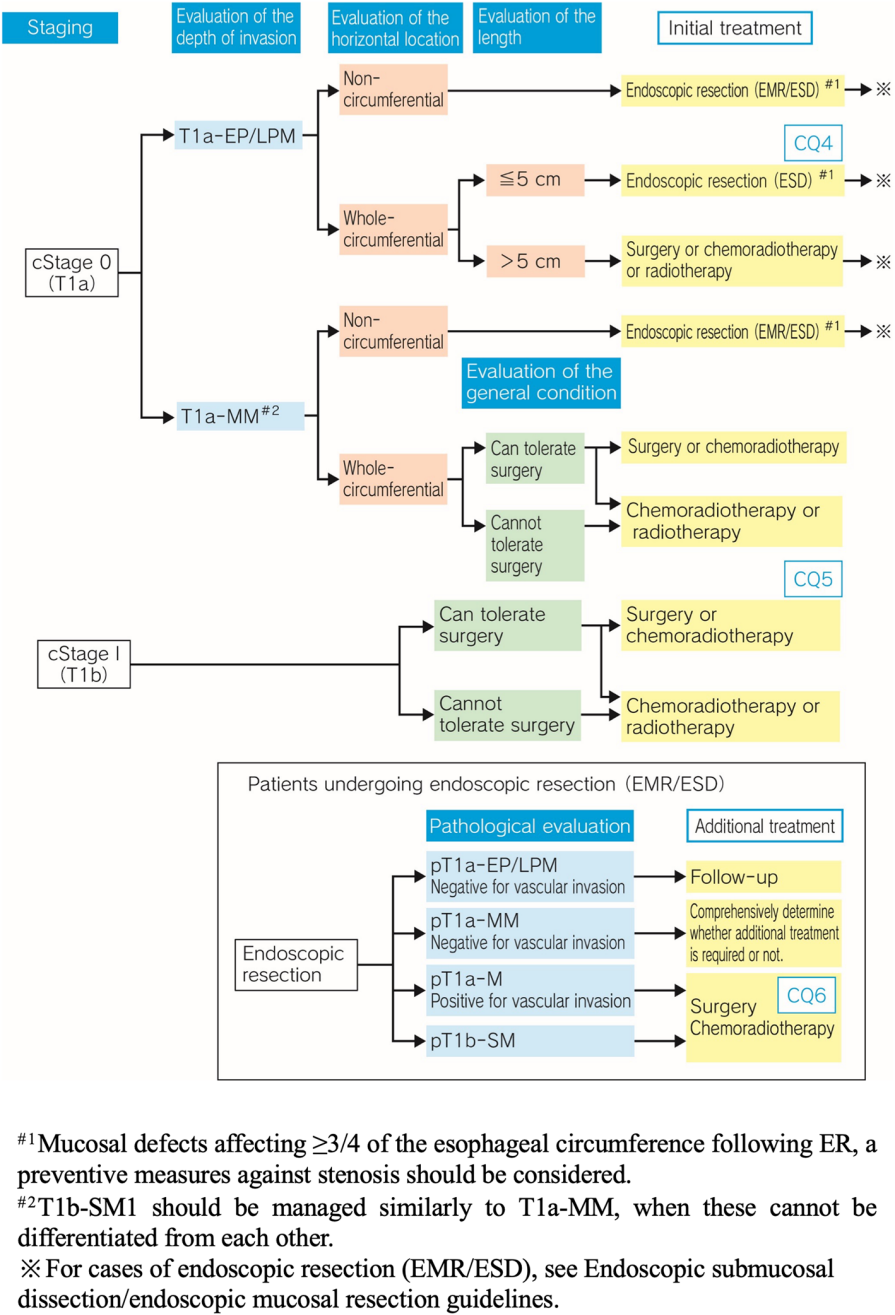
Treatment Algorithm for cStage 0 to I Esophageal Cancer

#### Summary

To determine the treatment strategy for cStage 0 or I carcinoma of the esophagus, the clinical stage of the disease should first be confirmed by modalities such as upper gastrointestinal endoscopy, computed tomography (CT) of the neck, chest and abdomen, and positron-emission tomography (PET). Then, it is important to assess the depth of tumor invasion to select the most appropriate treatment from among the options of endoscopic resection (ER), surgery, and chemoradiotherapy. The treatment algorithm for cStage 0/cStage I carcinoma of the esophagus is described in such a way as to make it consistent with the ESD/EMR guidelines for esophageal cancer.

Minimally invasive ER could be considered where the physician wavers in his/her assessment of the depth of tumor invasion and in patients who cannot tolerate surgery due to poor general condition. Assessment of the circumferential extent of the lesion should be undertaken in patients with clinical (c)Stage 0 disease (cT1a; cT1b-submucosal 1 [SM1] should be managed similarly to cT1a-muscularis mucosae [MM], when these cannot be differentiated from each other) scheduled to undergo ER to determine the risk of development of post-ER stenosis. When the post-resection ulcer is expected to involve ≥ 3/4th of the esophageal circumference, a preventive measures against stenosis should be considered, as such lesions are associated with a high risk of stenosis after ER. In patients with cStage I (T1b) disease, the selection between surgery and chemoradiotherapy should be made after assessing the patient’s surgical tolerability.

Post-ER pathological assessment is of vital importance to determine if any additional treatment might be required to ensure radical cure. In patients classified as having pathological (p)T1a-epithelium (EP)/lamina propria mucosae (LPM) disease, follow-up may be scheduled; on the other hand, in patients diagnosed as having pT1a-MM/pT1b-SM disease, additional treatment (surgery or chemoradiotherapy) should be considered.

**CQ4: What would be the recommendation for preventing postoperative stenosis following endoscopic resection of superficial cancer of the esophagus?**

### Recommendation statement

There is evidence to strongly recommend oral prednisolone treatment, submucosal triamcinolone injection, or concurrent oral prednisolone treatment + submucosal triamcinolone injection for preventing stenosis after endoscopic resection. (*Consensus rate: 85.2% [23/27], strength of evidence: C*).

### Explanatory note

A search of the literature in relation to this CQ yielded 255 PubMed articles, 52 Cochrane articles, and 107 ICHUSHI articles. Along with one additional article identified by a manual search, the 415 articles, in total, were subjected to a primary screening. From the primary screening, we extracted 42 articles referring to the following stenosis prevention measures used in clinical practice: “oral prednisolone treatment,” “submucosal triamcinolone injection,” “oral prednisolone treatment + submucosal triamcinolone injection,” “prophylactic balloon dilatation,” “triamcinolone acetonide-filling method,” “shielding with a polyglycolic acid sheet,” and “esophageal stenting (not approved in Japan)”. These articles were subjected to a secondary screening, and finally, 21 articles (3 randomized controlled studies, 3 non-randomized studies, and 15 observational studies) were selected for qualitative systematic reviews.

In the systematic reviews, non-circumferential resection and whole circumferential resection cases were examined separately, because the outcomes such as the “incidence of stenosis” and “number of dilatation sessions” differed greatly between the two procedures.

Table 2 shows the incidence of stenosis and the number of dilatation sessions in cases of post-ER ulcers involving ≥ 3/4th of the esophageal circumference, but not the entire esophageal circumference. The incidence of stenosis was 8.6–23.1% in patients treated with oral prednisolone [15–18], 9.2–36.2% in patients treated with submucosal triamcinolone injection [17–21], 10.0–13.3% in patients treated with oral prednisolone + submucosal triamcinolone injection [18, 19], and 50.0–80.0% in patients in whom no prophylactic measures were adopted [15–17, 19, 21]. Thus, adoption of preventive measures was associated with a reduced incidence of stenosis and lower number of dilatation sessions as compared with no adoption of preventive measures.

**Table 2:** Outcomes of prophylactic measures undertaken in cases of non-circumferential resection

Prophylactic measure	Circumferential extent	Incidence of stenosis	No. of dilatation sessions	Reference number
PSL	> 3/4^a^	23.1% (3/13)	3.0^b^	[15]
PSL	> 3/4	14.3% (2/14)	6.0^b^	[16]
PSL	> 3/4	20.0% (5/25)	–	[17]
PSL	> 3/4^a^	8.6% (6/70)	0^c^	[18]
TA	> 3/4^a^	33.3% (2/6)	–	[17]
TA	> 3/4	9.2% (8/87)	0^c^	[18]
TA	> 3/4	36.2% (17/47)	6.0^c,d^	[19]
TA	> 3/4	11.3% (13/115)	7.0^c^	[20]
TA	> 3/4	19.1% (4/21)	8.9^b^	[21]
PSL + TA	> 3/4	10% (1/10)	–	[18]
PSL + TA	> 3/4	13.3% (2/15)	5.5^c,d^	[19]
Control	> 3/4^a^	80.0% (8/10)	16.9^b^	[15]
Control	> 3/4	64.3% (9/14)	7.5^b^	[16]
Control	> 3/4^a^	50.0% (11/22)	–	[17]
Control	> 3/4	75.0% (15/20)	8.8^b^	[21]
Control	> 3/4	60.7% (17/28)	12.5^c^	[19]

*PSL* Oral prednisolone treatment, *TA* submucosal triamcinolone injection

^a^Including some cases of whole circumferential resection, ^b^average, ^c^median, ^d^only cases of post-resection ulcer involving ≥ 7/8th of the esophageal circumference

Table 3 shows the incidence of stenosis and number of dilatation sessions in cases of post-ER ulcers involving the entire esophageal circumference. The incidence of stenosis was 33.3–100% in patients treated with oral prednisolone [2, 4, 8, 16, 16, 22], 100% in patients treated with submucosal triamcinolone injection [18, 19, 23], 18.8–91.7% in patients treated with oral prednisolone + submucosal triamcinolone injection [18–20], and 100% in whom no prophylactic measures were adopted [16, 19, 22, 23]. In patients with whole-circumferential post-ER ulcers, submucosal triamcinolone injection alone was not effective for preventing stenosis, but the other prophylactic measures lowered the incidence of stenosis and decreased the number of dilatation sessions required as compared with the group in which no preventive measures were adopted.

**Table 3:** Outcomes of prophylactic measures undertaken in cases of whole circumferential resection

Prophylactic measure	Incidence of stenosis	No. of dilatation sessions	Reference number
PSL	33.3% (1/3)	2.0^a^	[16]
PSL	33.3% (8/24)	–	[18]
PSL	100% (10/10)	13.8^a^	[22]
TA	100% (4/4)	–	[18]
TA	100% (5/5)	10.4^a^	[23]
TA	100% (6/6)	–	[19]
PSL + TA	18.8% (3/16)	–	[18]
PSL + TA	71.4% (10/14)	–	[19]
PSL + TA	91.7% (11/12)	13.0^b^	[20]
Control	100% (2/2)	11.0^a^	[16]
Control	100% (5/5)	22.2^a^	[23]
Control	100% (5/5)	–	[19]
Control	100% (13/13)	33.5^a^	[22]

*PSL* Oral prednisolone treatment, *TA* submucosal triamcinolone injection

^a^Average, ^b^median

As for adverse events, 0.7% of patients treated with oral prednisolone (one of 134 patients) developed cytomegalovirus enterocolitis [18]. Perforation during balloon dilatation was observed in 1.9% of patients treated with submucosal triamcinolone injection (6 of 308 patients), which was only slightly higher than the incidence of 1.5% in the control group (in which no prophylactic measures were adopted; 1 of 69 patients) [19, 20, 23].

Thus, oral prednisolone treatment, submucosal triamcinolone injection, and concurrent oral prednisolone treatment + submucosal triamcinolone injection decreased the incidence of stenosis and number of dilatation sessions required as compared with no adoption of prophylactic measures, and were also associated with a very low incidence of adverse events. As for the burden on the patients, oral prednisolone treatment places some burden on the patients, because it takes 6–18 weeks, while submucosal triamcinolone injection places little burden on patients, because it is often performed only at the end of the ESD procedure. In regard to the costs, prednisolone costs about 10 yen per 5-mg tablet and the total cost for the usual treatment duration of 6–18 weeks is about 1500–4500 yen. Submucosal triamcinolone injection is inexpensive: each 50 mg/5 mL vial costs about 200 yen, and 1–3 vials (about 200–600 yen) are usually used. In addition, balloon dilatation used for the treatment of stenosis is expensive, corresponding to 12,480 medical remuneration points (37,440 yen for patients who pay 30% of the medical fees). The above-described data indicate that the number of dilatation sessions and total treatment cost can be reduced by using oral prednisolone treatment and/or submucosal triamcinolone injection. We concluded that oral prednisolone treatment, submucosal triamcinolone injection, or concurrent oral prednisolone treatment + submucosal triamcinolone injection can be recommended, because each of these was associated with a reduced incidence of stenosis and number of dilatation sessions required after endoscopic treatment of esophageal cancer, and also little risk. No comparison was made between oral prednisolone treatment and submucosal triamcinolone injection, because there were no informative articles.

In patients in whom prophylactic balloon dilatation was performed/not performed for the prevention of post-ER stenosis when they had no symptoms of stenosis, the incidence of stenosis was 58.6%/91.7%, and surprisingly, the number of dilatation sessions required for treatment was 8/4.5 [24]. There were no adverse events associated with prophylactic balloon dilatation. Prophylactic balloon dilatation decreased the incidence of stenosis [24], but it was less effective than oral prednisolone treatment and submucosal triamcinolone injection. In addition, the number of balloon dilatation sessions required for treatment was greater in patients who had received prophylactic balloon dilatation than in those who had not received any prophylactic measures, and prophylactic balloon dilatation increased the burden on the patients as well as the costs; therefore, we concluded that there is no evidence to recommend prophylactic balloon dilatation prior to the onset of symptoms of stenosis.

There were two reports identified on the triamcinolone acetonide-filling method. Although this procedure requires endoscopic examination every 2 weeks and additional triamcinolone acetonide filling, as needed, until epithelialization of the ulcer, it has been reported to be highly effective: the incidence of stenosis requiring balloon dilatation was only 4.5–5.0% and the number of dilatation sessions required for the treatment of stenosis was 2–3 [25, 26]. Although this method is promising, we concluded that currently, there is insufficient evidence to recommend it, because the two studies involved only a small number of patients (22 and 20 patients) and most of them were from single-institute.

Three articles were extracted on shielding with a polyglycolic acid sheet [27–29]. One of the articles reported the usefulness of combined use of a polyglycolic acid sheet with stenting, which is not approved in Japan. The other two studies compared shielding with a polyglycolic acid sheet and submucosal triamcinolone injection [28] and no prophylactic measures [29], and found no significant difference in the incidence of stenosis. Although shielding with a polyglycolic acid sheet was not associated with adverse events, it is expensive: the sheet costs about 8000 yen and the physiological tissue adhesive that fixes the sheet to the ulcer floor costs about 54,000 yen. We concluded that there is no evidence to recommend shielding with a polyglycolic acid sheet, because of the inferior risk–benefit balance as compared to oral prednisolone treatment or submucosal triamcinolone injection.

Three overseas studies have reported the use of esophageal stents, which are not yet approved in Japan, to prevent benign stenosis [30–32]. All these studies used a covered metal stent, which was inserted into the ulcer soon after endoscopic treatment and removed 2–8 weeks later. The incidence of stenosis was 18.2% (2 of 11 cases) in cases of post-resection ulcers involving ≥ 3/4th of the esophageal circumference [32], and the outcomes of esophageal stenting were comparable to those of oral prednisolone treatment or submucosal triamcinolone injection as prophylactic measures against the development of stenosis. On the other hand, rather promising outcomes were reported in cases of post-resection ulcers involving the entire esophageal circumference: the incidence of stenosis was 17.4% (4 of 23 cases) in one study [30] and 50% (6 of 12 cases) in another. However, it should be noted that the incidence of adverse events was also higher (15.2%, 7 of 46 cases; stent migration in 5 cases, bronchial fistula in 1 case, and pain in 1 case) as compared with that associated with other prophylactic modalities, and some of the adverse events were serious.

All of the aforementioned studies, except one of oral prednisolone treatment versus submucosal triamcinolone injection, were non-randomized studies. We concluded that the strength (certainty) of the overall evidence for all outcomes is C, because use of oral prednisolone treatment or submucosal triamcinolone injection was highly effective as compared with no adoption of prophylactic measures, although the risk of bias is high. However, we concluded that oral prednisolone treatment or submucosal triamcinolone injection is strongly recommended, because all other factors, such as the risk–benefit balance, patient preferences, and costs support to adoption of oral prednisolone treatment or submucosal triamcinolone injection as a prophylactic measure to prevent stenosis after ER.

**CQ5: Which is recommended, esophagectomy or definitive chemoradiotherapy, in patients with cStage I (T1bN0M0) thoracic esophageal cancer?**

### Recommendation statement

There is weak evidence to recommend esophagectomy in patients with cStage I (T1bN0M0) thoracic esophageal cancer, and there is also weak evidence to recommend definitive chemoradiotherapy with adequate follow-up and salvage therapy in patients with cStage I who desire for esophageal preservation. (*Rate of consensus: 92.3% [24/26], strength of evidence: C*).

### Explanatory note

A search of the literature conducted to respond to this CQ yielded 347 PubMed articles, 64 Cochrane articles, and 192 ICHUSHI articles. These articles, together with 1 article published in 2021 and 1 article on the results of the JCOG0502 Study presented at the American Society of Clinical Oncology (ASCO) Gastrointestinal Cancer Symposium (GI), were subjected to primary and secondary screenings, and 1 report of a meta-analysis [33], 3 reports of retrospective studies [34–36], and 1 report of a prospective study (JCOG0502) [37] were extracted. Of the references cited for data on cStage I esophageal cancer in the meta-analysis, 1 report of a study with a short follow-up period [38] was excluded, and the remaining 4 articles [39–42] were added, and a total of 8 articles were subjected to a qualitative systematic review. There was no published randomized controlled study directly comparing the outcomes of surgery and definitive chemoradiotherapy for cStage I cases.

The qualitative systematic review showed that the 5-year overall survival rate (odds ratio [OR], 0.68; 95% confidence interval [CI], 0.49–0.95; *p* = 0.02) and 5-year progression-free survival rate (OR 0.53; 95% CI 0.30–0.95; *p* = 0.03) were significantly higher in patients treated by surgery. However, the level of evidence is low, because none of the studies was a randomized controlled study; therefore, the background factors of the patients differed among many studies.

According to the only report of a prospective study, JCOG0502, patients treated by surgery showed a 5-years progression-free survival rate of 81.7% (95% CI 75.7–86.3), and a 5-year overall survival rate of 86.5% (95% CI 81.0–90.5), while patients treated by definitive chemoradiotherapy showed a complete response rate of 87.3% (95% CI 81.1–92.1), a 5-year progression-free survival rate of 71.6% (95% CI 63.9–78.0), a 5-year overall survival rate of 85.5% (95% CI 78.9–90.1), and a 5-year esophagus preservation rate of 80.4% (95% CI 73.3–85.8). Although surgery significantly prolonged the progression-free survival (hazard ratio [HR]: 1.478 [95% CI 1.01–2.16]), there was no significant difference in the overall survival between the two treatment modalities (HR 1.05 [95% CI 0.67–1.64]). In regard to the toxicity (according to the Common Terminology Criteria for Adverse Events [CTCAE]), the acute toxicities in the patients receiving definitive chemoradiotherapy in the JCOG0502 Study were esophagitis (Grade 3–4; 10%), leukopenia (Grade 3–4; 11%), and febrile neutropenia (Grade 3–4; 1.9%), and the late toxicities were esophagitis (Grade 3–4; 0.6%), radiation pneumonitis (Grade 3–4; 1.9%), and pleural effusion (Grade 3–4; 2.5%). No deaths were reported during or within 30 days of completion of the protocol treatment. On the other hand, the postoperative complications reported in the patients who were treated by surgery were pneumonia (Grade ≥ 2; 13%), recurrent laryngeal nerve paralysis (Grade ≥ 2; 15%), and anastomotic leakage (Grade ≥ 2; 15%); in addition, two cases of treatment-related death were reported. Although patients treated by chemoradiotherapy showed a shorter progression-free survival than those treated by surgery, patients who received chemoradiotherapy followed by appropriate aftertreatment showed a comparable overall survival to those who were treated by surgery, with a 5-year esophagus preservation rate of 80.4%. Accordingly, chemoradiotherapy is a valid treatment option for patients who wish for their esophagus to be preserved.

On the basis of the systematic review of the extracted articles, we determined that surgery can be weakly recommended based on the survival rates; however, taking into account the risk–benefit balance and patient preferences, we concluded that “there is weak evidence to recommend esophagectomy in patients with cStage I (T1bN0M0) thoracic esophageal cancer, and there is also weak evidence to recommend chemoradiotherapy with adequate follow-up and salvage therapy in patients who wish to have their esophagus preserved”.

**CQ6: Which is recommended as an additional treatment—esophagectomy or chemoradiotherapy—in cases with a pT1a-MM lesion showing positive vascular invasion or a pT1b-SM lesion following endoscopic treatment for superficial esophageal cancer?**

### Recommendation statement

There is evidence to recommend esophagectomy or chemoradiotherapy as an additional treatment in patients identified as having a pT1a-MM lesion with positive vascular invasion or a pT1b-SM lesion after endoscopic treatment for superficial esophageal cancer; however, currently there is insufficient evidence to definitively recommend one over the other. (*Rate of consensus: 89.3% [25/28]; strength of evidence: C*).

### Explanatory note

A search of the literature conducted to respond to this CQ yielded 23 PubMed articles, 2 Cochrane articles, and 8 ICHUSHI articles through a primary screening, and secondary screening of these articles led to the retrieval of 14 articles, which were subjected to a qualitative systematic review.

All of the 14 articles were reports of retrospective studies, including three studies of only cases treated by surgery as an additional treatment after endoscopic resection, four studies of only cases treated by chemoradiotherapy, and seven studies comparing surgery and chemoradiotherapy as an additional treatment. There was a bias in the patient characteristics, such as a preferential selection of chemoradiotherapy for elderly patients and for patients who could not tolerate surgery, and differences in the radiation dose according to the depth of invasion and the presence/absence of vascular invasion among the studies [43–54].

In patients who were treated by surgery and chemoradiotherapy, the 5-year overall survival rates were 79–100% and 60–100%, respectively, and the 5-year disease-free (recurrence-free) survival rates were 89.5–100% and 55–100%, respectively. The reported risk factors for recurrence include SM invasion, positive vascular invasion, and tumor diameter ≥ 40 mm. In the pT1a-MM and pT1b-SM cases overall, the overall survival and disease-free (recurrence-free) survival rates tended to be higher in patients who were treated by surgery, although the difference was not significant. Three studies compared surgery and chemoradiotherapy in cases with SM or deeper invasion, and all three reported a significantly worse prognosis in patients who were treated by chemoradiotherapy as compared with that in patients treated by surgery. In the JCOG0508 Study, cT1bN0 esophageal cancer with a limited depth of invasion, which was considered endoscopically resectable, was treated endoscopically, and patients with pathologically confirmed complete resection who had pT1a with positive vascular invasion or pT1b received additional chemoradiotherapy. With such treatment, these patients showed a 3-year survival rate of 90.7%, suggesting the usefulness of the strategy [55]. On the other hand, of the 15 patients who had positive vertical resection margins after endoscopic treatment and received definitive chemoradiotherapy, 4 had metastatic recurrence and 3 (20%) died of the disease. Further investigation is needed to determine the most suitable additional treatment(s) for cases in which complete resection fails to be accomplished by endoscopic treatment.

As for the toxicity of surgery and chemoradiotherapy, there are few systematic reviews of postoperative complications following additional surgery after endoscopic resection or adverse events associated with chemoradiotherapy as additional treatment. Therefore, we investigated the toxicity of surgery and chemoradiotherapy by referring to reports of initial surgery for cT1 esophageal cancer and additional chemoradiotherapy after endoscopic resection, regardless of the histopathological results. The treatment-related mortality due to surgical complications in T1 cases has been reported to be 0.2–3.6%. The reported causes of treatment-related death after additional chemoradiotherapy were radiation pneumonitis, sudden death, and myocardial infarction. In addition, esophageal fistula (3.2%), esophagostenosis (3.2%), Grade 3 cardiac ischemia (1%), and respiratory failure (2.8%) were reported as late complications.

In summary, although there are no randomized controlled studies or prospective studies directly comparing surgery and chemoradiotherapy as additional treatments after endoscopic resection, the therapeutic responses (in terms of the overall survival) to surgery and chemoradiotherapy appear to be comparable in pT1a-MM and pT1b cases. However, even in pT1b (particularly SM2-3), pT1a-MM, or pT1b-SM1 cases, patients with positive vascular invasion have been reported to show a tendency towards a worse prognosis after chemoradiotherapy than after surgery, suggesting that surgery may be the optimal additional treatment option in these high-risk patients.

Thus, it was rather difficult to provide a clear and definitive response to this CQ on the ground of the evidence obtained from this systematic review. Any treatment is associated with some risk of postoperative complications and adverse events, and treatment-related deaths have also been reported. Therefore, treatment should be carefully tailored to individual cases, after taking into consideration the risk–benefit balances and patient preferences.

## Treatment algorithm for cStage II to Stage III esophageal cancer (Fig. 2)

**Fig. 2:**
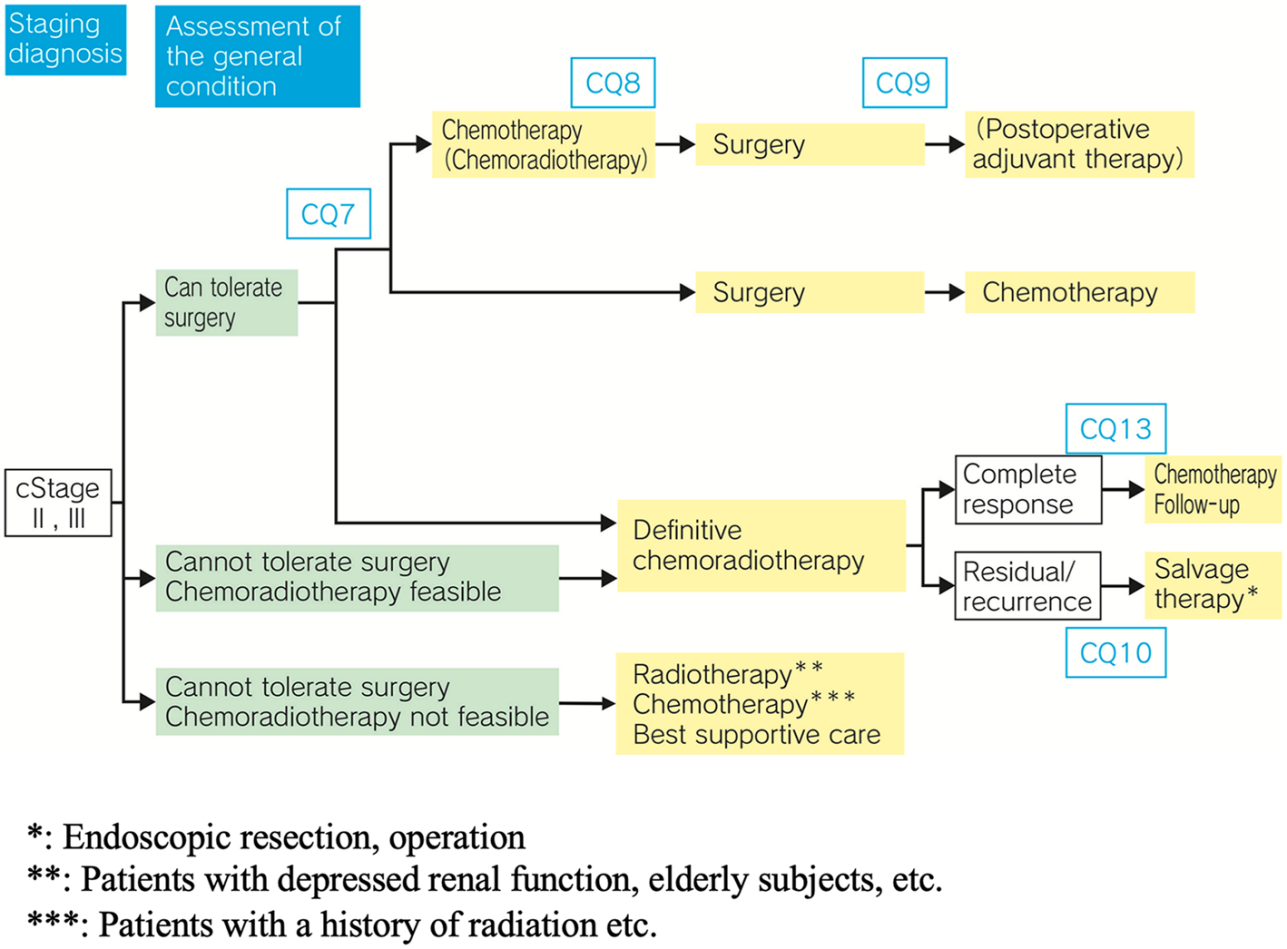
Treatment Algorithm for cStage II to III Esophageal Cancer

### Summary

To select the appropriate treatment strategy for cStage II and III esophageal carcinoma, the patient’s tolerability to surgical intervention should first be confirmed through evaluation of the patient’s general condition after accurate diagnosis of the clinical stage by upper gastrointestinal endoscopy, CT, and PET. When no problem is identified with respect to the surgical tolerability, preoperative chemotherapy followed by radical resection should be considered as the first-line therapy. Radical resection without preoperative treatment or with preoperative chemoradiotherapy could also be selected. In cases of surgery without any preoperative treatments, administration of adjuvant chemotherapy should be considered in accordance with the histopathologic diagnosis confirmed in the resected specimens (especially for lymph node metastasis-positive cases). Recently, nivolumab was reported to be useful as adjuvant chemotherapy in patients in whom R0 resection was achieved following preoperative chemoradiotherapy. Definitive chemoradiotherapy (≥ 50 Gy) should be considered in patients who are unable to tolerate surgery, refuse surgery, or wish to receive esophagus-preserving therapy as first-line therapy, in whom chemoradiotherapy is feasible. Patients in whom complete response is achieved should be followed up, and in case of a residual or recurrent lesion, the practicability of surgical resection or endoscopic resection as salvage therapy should be explored. In patients unable to tolerate surgery who are not suitable candidates for chemoradiotherapy either, radiation therapy (e.g., in patients with depressed renal function, elderly subjects), chemotherapy (e.g., in patients with a history of radiation), or best supportive care should be considered.

**CQ7: What should be recommended—primarily surgery, or definitive chemoradiotherapy—in patients with cStage II or III esophageal cancer?**

### Recommendation statement

There is weak evidence to recommend primarily surgery for patients with cStage II or III esophageal cancer. (*Rate of consensus: 100% [28/28], strength of evidence: C*).

### Explanatory note

A search of the literature conducted to respond to this CQ yielded 363 PubMed articles, 56 Cochrane articles, and 241 ICHUSHI articles, which were subjected to primary and secondary screenings. Finally, 28 papers, consisting of 3 reports of randomized controlled studies, 22 reports of observational studies, and 3 systematic reviews were retrieved, which were subjected to a qualitative systematic review.

There are three reports of randomized controlled studies directly comparing the results of surgery and definitive chemoradiotherapy [56–58]. Because all these reports are from overseas, however, they differed greatly in the therapy regimens and treatment policies adopted as compared with those adopted in Japan. In regard to observational studies, 22 reports of studies comparing surgery and definitive chemoradiotherapy in patients with cStage II or III esophageal cancer, including 10 papers from Japan, were retrieved [36, 38, 42, 59–76]. None of the studies was a randomized controlled study, so that the background factors of the patients differed among studies, and the therapeutic regimens adopted were different from those currently adopted as standard treatments in Japan. As for the survival time, 10 of the 22 papers indicated that surgery yielded a significant prolongation of the overall survival time. Only one paper indicated prolongation of the overall survival time following definitive chemoradiotherapy. Of the three systematic reviews, one reported a significant prolongation of the overall survival time in patients treated by surgery, while the remaining two showed no significant difference in the overall survival between the two modalities [77–79]. Thus, it was rather difficult to provide a clear and definitive response to this CQ on the ground of the evidence obtained from the systematic review.

In regard to the toxicity, the late toxicities reported in the patients who received definitive chemoradiotherapy in the JCOG9906 Study were esophagitis (Grade 3–4; 13%), pericardial effusion (Grade 3–4; 16%), pleural effusion (Grade 3–4; 9%), and radiation pneumonitis (Grade 3–4; 4%); death of four cases was also reported [80]. Out of the 22 observational studies, 6 from Japan reported operation-related deaths at an incidence rate of 0–4% among patients treated by surgery. In the JCOG9907 Study, there were two cases of operation-related death among 330 cases. It should be noted that there is a potential risk of serious complications among patients receiving definitive chemoradiotherapy as well as among those treated by surgery [81].

The basis for considering that surgery may yield greater improvement of the overall survival rate as compared to definitive chemoradiotherapy is thus rather tenuous, and both treatment modalities entail a significant risk of toxicity. However, the 5-years survival rate obtained with preoperative chemotherapy plus surgery in the JCOG9907 Study was 55%, as compared to 37% in the JCOG9906 Study. The JCOG1406A Study, which is a sub-study comparing the JCOG9906 and JCOG9907 studies, reported that surgery significantly improved the overall survival rate as compared with definitive chemoradiotherapy (HR 1.72 [95% CI 1.19–2.50]) [80, 81]. The JCOG1109 Study, the results of which were published in January 2022, showed that preoperative docetaxel + cisplatin + 5-fluorouracil (5-FU) (DCF) therapy prolonged the survival as compared with preoperative cisplatin + 5-FU (CF) therapy, which had been the standard treatment (3-year survival rate: preoperative CF therapy, 62.6%; DCF therapy, 72.1%) (HR: 0.68 [95% CI 0.50–0.92]) [82]. In addition, many observational studies conducted in Japan have revealed more gratifying results in groups treated by surgery. Therefore, we concluded that “there is strong evidence to recommend preoperative triplet chemotherapy with docetaxel + cisplatin + 5-FU plus surgery for the treatment of cStage II or III esophageal cancer”.

The JCOG0909 Study assessed the usefulness of preoperative definitive chemoradiotherapy followed by positive surgical intervention as salvage operation in patients with cStage II or III esophageal cancer [83]. In the JCOG0909 Study, a three-dimensional treatment plan and multiple-field irradiation were introduced and the fractional and total radiation doses were modified to 1.8 Gy and 50.4 Gy, respectively, in an attempt to reduce the risk of adverse events and the risk associated with salvage esophagectomy that was seen in the JCOG9906 Study. According to the report of the final analysis, 2 of the 96 patients enrolled in the study were excluded from the analysis, and 55 patients (59%) had a complete response. The 5-years overall survival rate was 64.5% (95% CI 53.9–73.3), the 5-year recurrence-free survival rate was 48.3% (95% CI 37.9–58.0), and the 5-year esophagus preservation rate was 54.9% (95% CI 44.3–64.4). Grade 3 or higher late toxicity was observed in 8 patients (8.5%). Twenty-five patients (26.0%) underwent salvage surgery (esophagectomy in 20 cases and lymph node dissection alone in 5 cases), and of these patients, 5 (20.0%) developed grade 3–4 surgery-related complications and there was 1 case of surgery-related death. However, R0 surgery could be achieved in 76.0% of the patients, suggesting that salvage surgery could be an effective treatment option if the indication is carefully selected. The results of the JCOG0909 Study suggest that definitive chemoradiotherapy at the dose of 50.4 Gy is one of the valid treatment options for patients with cStage II or III esophageal cancer who do not wish to undergo surgery as the primary treatment.

Both surgery and definitive chemoradiotherapy as primary treatment are covered by the national health insurance, and taking into consideration the risk–benefit balance, strength of evidence, and patient preferences, we concluded to state that there is weak evidence to recommend surgery as the primary treatment for patients with cStage II or III esophageal cancer.

**CQ8: Which is recommended—preoperative chemotherapy or preoperative chemoradiotherapy—in cStage II or III esophageal cancer patients scheduled to receive surgery as the primary treatment?**

### Recommendation statement

In patients with cStage II or III esophageal cancer who are scheduled to receive surgery as the primary treatment, there is strong evidence to recommend preoperative triplet chemotherapy with docetaxel + cisplatin + 5-FU. (*Rate of consensus: 84% [21/25], strength of evidence: A*).

### Explanatory note

In patients with cStage II or III esophageal cancer who are scheduled to receive surgery as the primary treatment, preoperative chemotherapy with cisplatin and 5-FU + surgery is recommended as the standard treatment in Japan, based on the results of the JCOG 9907 Study [81]. However, the optimal preoperative adjuvant therapy regimen has remained controversial, and numerous discussions have been held on the appropriateness of intense chemotherapy regimens and of chemotherapy combined with radiation therapy.

A search of the literature conducted to provide a response to this CQ yielded 278 PubMed articles, 47 Cochrane articles, and 293 ICHUSHI articles, which were subjected to primary and secondary screenings. Finally, six reports of randomized controlled studies and three reports of observational studies were retrieved and subjected to a qualitative systematic review.

There were three reports of randomized controlled studies [84–86] and three reports of observational studies [87–89] from overseas, comparing the results of preoperative chemotherapy + surgery and preoperative chemoradiotherapy + surgery. None of the studies found a significant difference in the 5-year survival rate between preoperative chemotherapy + surgery and preoperative chemoradiotherapy + surgery. There are 4 reports of randomized controlled studies from overseas comparing the adverse events of preoperative chemotherapy + surgery versus preoperative chemoradiotherapy + surgery. Although the surgery-related and non-surgery-related mortality rates and the mortality rate within 90 days of surgery did not differ significantly between the two modalities [86, 90], the incidence of severe adverse events was significantly higher in the preoperative chemoradiotherapy + surgery group [90], and odynophagia after preoperative treatment, impaired cardiac function after surgery, and symptoms that lower the quality of life (QOL), such as cough, were significantly more common in the preoperative chemoradiotherapy + surgery group [91, 92]. However, these studies also included many patients with esophageal adenocarcinoma or carcinoma of the esophagogastric junction, neither of which is common in Japan, and used different chemotherapy regimens/doses and radiation doses from those that are currently adopted as current standard treatment in Japan. Therefore, it is considered inappropriate to extrapolate these results to answer the CQ.

In Japan, the results of the JCOG1109 Study comparing 3 regimens of preoperative adjuvant therapy in patients with cStage II or III esophageal cancer scheduled to undergo surgery were presented in January 2022 [82, 93], based on which we arrived at a conclusion to respond to this CQ (presented at ASCO-GI 2022). The JCOG1109 Study is a phase III randomized controlled study comparing doublet preoperative chemotherapy with cisplatin + 5-FU (preoperative CF group), which is the current standard treatment, and preoperative triplet chemotherapy with docetaxel + cisplatin + 5-FU (preoperative DCF group), and between CF and preoperative chemoradiotherapy with CF + radiation at 41.4 Gy (preoperative CF-RT group). DCF yielded a significant prolongation of the overall survival time as compared with CF, and the incidence of perioperative complications remained acceptable in the DCF group. On the other hand, the study failed to show the superiority of CF-RT over CF in terms of the overall survival [82].

Both preoperative chemotherapy and preoperative chemoradiotherapy are covered by the national health insurance, and taking into consideration the risk–benefit balance, strength of evidence, and patient preferences, we concluded that in patients with cStage II or III esophageal cancer who are scheduled to receive surgery as the primary treatment, there is strong evidence to recommend preoperative triplet preoperative chemotherapy with docetaxel + cisplatin + 5-FU. However, the JCOG1109 Study included only patients aged up to 75 years of age from institutions that manage many patients with esophageal cancer and participate in Japan Clinical Oncology Group (JCOG) studies; therefore, the current standard treatment, i.e., preoperative doublet chemotherapy with cisplatin + 5-FU, will continue to remain a treatment option for elderly patients and those with comorbidities who are considered unable to tolerate triplet chemotherapy and for institutions with little experience in the use of triplet chemotherapy. In addition, the significance of preoperative chemoradiotherapy in patients with borderline resectable (br) T3 disease could not be excluded, and preoperative treatment combined with radiation should also be considered, according to the needs of individual cases.

**CQ9: Is postoperative adjuvant therapy recommended in cStage II or III esophageal cancer patients who have undergone preoperative adjuvant therapy plus surgery?**

### Recommendation statement

1. In patients with cStage II or III esophageal cancer who failed to show a pathologic complete response after preoperative chemoradiotherapy plus surgery with radical resection, there is strong evidence to recommend postoperative nivolumab therapy, regardless of the histologic type or tumor expression level of programmed death ligand 1 (PD-L1). (*Rate of consensus: 81% [21/26], strength of evidence: A*)
2. In patients with cStage II or III esophageal cancer who have undergone preoperative chemotherapy plus surgery with radical resection, but failed to achieve a pathologic complete response, there is currently no evidence to recommend postoperative nivolumab therapy. (*Rate of consensus: 92% [24/26], strength of evidence: D*)

### Explanatory note

A search of the literature to provide a response to this CQ yielded 208 PubMed articles, 31 Cochrane articles, and 101 ICHUSHI articles, along with 2 additional articles through primary screening; secondary screening of these articles led to the retrieval of 3 reports of randomized controlled studies [81, 94, 95], which were then subjected to a qualitative systematic review.

Preoperative chemotherapy plus surgery is currently the standard treatment for cStage II or III thoracic esophageal squamous cell carcinoma in Japan, as, while surgery plus postoperative chemotherapy was demonstrated to be superior to surgery alone in the JCOG9204 Study, the JCOG9907 Study demonstrated the superiority of preoperative chemotherapy over postoperative chemotherapy. There was only one report of a randomized controlled study pertinent to this CQ, which was from overseas [95]. According to this randomized controlled study, the 5-years recurrence-free survival rate was 35.0% in patients who received postoperative adjuvant chemotherapy after preoperative chemotherapy plus radical surgery, and 19.1% in a matched group that did not receive postoperative adjuvant chemotherapy (HR 0.62, *p* < 0.001) [95]. However, the report contains no detailed descriptions of randomization, blinding, or preoperative staging of the disease, and furthermore, the surgical procedures and chemotherapy described in the report differed from those used in Japan; therefore, we consider that the results of this study are not directly applicable to the clinical practice setting in Japan. Since the randomized controlled studies of preoperative/postoperative chemotherapy reported from Europe have been performed in patients with esophageal adenocarcinoma [96, 97], it would be inappropriate to extrapolate the results of those studies to answer this CQ.

In 2021, the CheckMate 577 study provided new evidence on the usefulness of nivolumab as postoperative chemotherapy [98]. This was an international phase III randomized controlled study conducted in Japan and other countries in patients with cStage II or III esophageal cancer or carcinoma of the esophagogastric junction in whom R0 resection was achieved following preoperative chemoradiotherapy. The patients enrolled in the study were randomized 2:1 to receive nivolumab or placebo (a total of 794 patients consisting of 532 in the nivolumab group and 262 in the placebo group), and the disease-free survival (primary endpoint of the study) was 22.4 months (95% CI 16.6–34.0 months) in the nivolumab group and 11.0 months (95% CI 8.3–14.3 months) in the placebo group; thus, the results demonstrated the superiority of nivolumab with statistically significance (HR 0.69, 96.4% CI 0.56–0.86, *p* < 0.001).

While deciding to recommend nivolumab in the guidelines, we wish to make a note of the following points in the CheckMate 577 study: (1) The efficacy and safety of nivolumab exclusively in the Japanese population was not reported; (2) the efficacy and safety of nivolumab following preoperative chemotherapy was not established; (3) the efficacy of nivolumab in patients who showed pathologic complete response to preoperative therapy was not established; and (4) no data on the overall survival were shown, and the long-term efficacy, including the therapeutic response after recurrence remains to be established. However, the usefulness of nivolumab as postoperative chemotherapy in the overall subject population was demonstrated, and furthermore, nivolumab was well-tolerated, with an acceptable incidence of adverse events.

Therefore, taking into consideration the risk–benefit balance, our recommendations on postoperative chemotherapy are as follows:

1. In patients with cStage II or III esophageal cancer who have undergone preoperative chemoradiotherapy plus surgery with radical resection, but failed to achieve a pathologic complete response, there is strong evidence to recommend postoperative nivolumab therapy, regardless of the histologic type or tumor expression level of programmed death ligand 1 (PD-L1). (*Rate of consensus: 81% [21/26], strength of evidence: A*).
2. In patients with cStage II or III esophageal cancer who have undergone preoperative chemotherapy plus surgery with radical resection, but failed to achieve a pathologic complete response, there is currently no evidence to recommend postoperative nivolumab therapy. (*Rate of consensus: 92% [24/26], strength of evidence: D*).

**CQ11: Is salvage surgery recommended for residual or recurrent lesions after chemoradiotherapy in patients with untreated resectable esophageal cancer?**

### Recommendation statement

There is weak evidence to recommend salvage surgery for residual or recurrent lesions after chemoradiotherapy in patients with untreated resectable esophageal cancer. (*Rate of consensus: 96.4% [27/28], strength of evidence: C*).

### Explanatory note

There are limited treatment options for residual or recurrent lesions after definitive chemoradiotherapy. Although salvage surgery has been reported to be the only radical treatment for resectable lesions, it has a high perioperative mortality rate, and its usefulness has not been established.

A search of the literature to provide a response to this CQ yielded 337 PubMed articles, 144 Cochrane articles, and 100 ICHUSHI articles, which were subjected to primary and secondary screenings, leading to the retrieval of 6 reports of retrospective studies [99–104]. Examination of the 30-day mortality as a short-term outcome, and of the 3-year and 5-year survival rates as long-term outcomes resulted in the exclusion of two papers [103, 104]. The remaining four papers [99–102], along with the results of the JCOG0909 Study [83]. Two of the studies compared salvage surgery after definitive chemoradiotherapy and scheduled surgery after preoperative chemoradiotherapy (using propensity score matching in one study). One of the studies compared salvage chemoradiotherapy and salvage surgery. The remaining two studies analyzed only salvage surgery. None of the studies is very directly relevant to the CQ, and there are no reports of randomized controlled studies comparing salvage surgical therapy and non-surgical therapy. Therefore, we investigated the outcomes of salvage surgery for residual or recurrent lesions after definitive chemoradiotherapy.

All studies comparing salvage surgery after definitive chemoradiotherapy and scheduled surgery after preoperative chemoradiotherapy were from overseas. According to these reports, there were no significant differences in the 30-days mortality rate after salvage surgery (3.1–11.4%) and scheduled surgery following preoperative chemoradiotherapy (4.6–8.4%) or in the 3-years survival rate after salvage surgery (20–48%) and scheduled surgery following preoperative chemoradiotherapy (43.4–55%).

According to the JCOG9906 Study, which investigated the usefulness of definitive chemoradiotherapy in patients with untreated resectable esophageal cancer (cStage II or III, excluding T4) who did not wish to undergo surgery as initial treatment in Japan, the median survival was 29 months, the 3-years overall survival rate was 44.7%, and the 5-years overall survival rate was 36.8%. Seventy-six patients were enrolled, and ten of the patients (13.2%) eventually underwent salvage surgery for residual or recurrent lesions. The patients who underwent salvage surgery showed a median survival of 16.7 months and a 3-year overall survival rate of 40% [80]. The JCOG0909 Study also included patients with cStage II or III (non-T4) esophageal cancer but planned to perform salvage surgery for residual or recurrent lesions. Ninety-six patients were enrolled, and 27 of the 94 patients analyzed (28.7%) underwent salvage surgery for residual or recurrent lesions, with R0 resection achieved in 19 patients (76.0%). Five of the patients who underwent salvage surgery (20.0%) developed ≥ Grade 3 complications and 1 patient (4.0%) died in the perioperative period. The salvage surgery yielded gratifying results, with a 3-year overall survival rate (primary endpoint) of 74.2%, indicating the usefulness of definitive chemoradiotherapy for patients with resectable esophageal cancer in whom salvage surgery is planned for residual or recurrent lesions.

When residual or recurrent lesions are found after definitive chemoradiotherapy, salvage surgery is the only radical treatment for resectable lesions. In radiotherapy in definitive chemoradiotherapy, three-dimensional treatment planning allows optimization of the doses to the tumor and risk organs, enabling highly accurate treatment, and the optimal total dose and irradiation area are determined highly accurately. In regard to salvage surgery for residual or recurrent lesions, improvements in the operative procedures and advances in perioperative management have reduced the incidence of perioperative complications and the perioperative mortality. We concluded that there is weak evidence to recommend salvage surgery for residual or recurrent lesions after chemoradiotherapy in patients with untreated resectable esophageal cancer, while it is necessary to give careful consideration to the safety of the surgery and perioperative managements, and to obtain the patient’s informed consent after providing an explanation of the risks of the surgery.

## Treatment algorithm for cStage IVA esophageal cancer (Fig. 3)

**Fig. 3:**
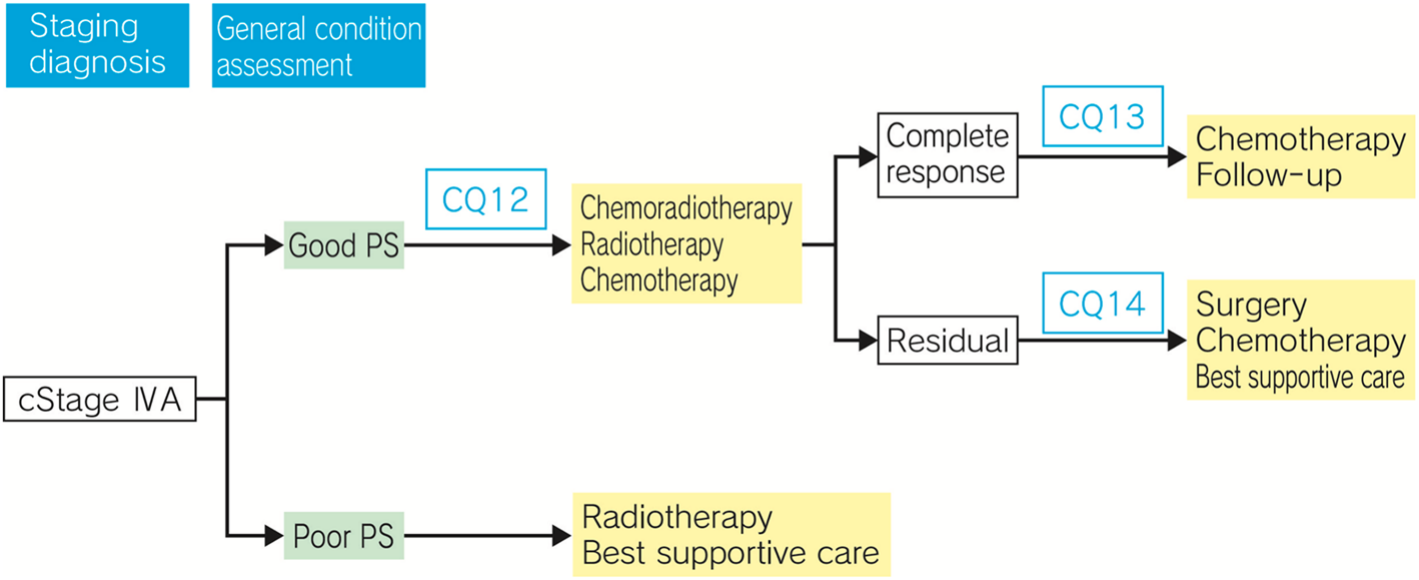
Treatment Algorithm for cStage IVA Esophageal Cancer

### Summary

Although cStage IVA esophageal cancer is unresectable, the lesion is localized, and chemoradiotherapy is expected to provide cure, if it is well-tolerated by the patient. In chemoradiotherapy, attention should be paid not only to adverse events during treatment, but also to late adverse events after completion of treatment. The possible need of salvage surgery for residual or recurrent lesions after chemoradiotherapy may entail an increase in the risk of operation-related death; therefore, it is necessary to judge the situation comprehensively with due consideration given to the risk–benefit balance, based on tumor status, patient’s condition, and other available treatment options.

In patients who cannot tolerate chemoradiotherapy, radiation alone should be considered, but it has been reported to be inferior to chemoradiotherapy in terms of therapeutic response.

**CQ12: Is chemoradiotherapy recommended for unresectable cStage IVA esophageal cancer?**

### Recommendation statement

There is only weak evidence to recommend definitive chemoradiotherapy for the treatment of unresectable cStage IVA esophageal cancer. (*Rate of consensus: 100% [28/28]; strength of evidence: C*).

### Explanatory note

A search of the literature to provide a response to this CQ yielded 271 PubMed articles, 22 Cochrane articles, and 147 ICHUSHI articles, along with additional 4 other papers. Ninety-three papers were retrieved through primary screening, and 7 papers were extracted through secondary screening and subjected to a qualitative systematic review. There were no reports addressing the population-intervention-comparison-outcome (PICO) question consistent with this CQ, and 4 reports of phase I/II studies of definitive chemoradiotherapy, 1 report of a phase II study of induction chemotherapy, and 2 reports of randomized controlled studies involving chemoradiotherapy were retrieved.

Definitive chemoradiotherapy is one of the treatment options that could offer an opportunity for cure in patients with unresectable, locally advanced esophageal cancer. However, fatal complications (e.g., tumor perforation, penetration) could also occur in association with a favorable response to chemoradiotherapy. In Japan, in view of the results of the JCOG0303 Study, definitive chemoradiotherapy is often selected for the treatment of patients with unresectable, locally advanced esophageal cancer with a good PS. The validity of this treatment modality was examined by comparison of the percentage of cases showing long-term survival (merit) and the incidence of fatal complications (demerit).

Although there is a paucity of data on the long-term survival, the 2- or 3-years survival rate is reported to be in the range of about 20–30% [105–107], and the percentage of patients showing long-term survival is estimated to be about 15–20%. The patient populations in these studies included a certain proportion of patients with a performance status (PS) score of 2, and a common feature of these studies was that the prognosis was unfavorable in patients with factors related to a poor PS, such as “weight loss from the usual weight,” suggesting the possibility that a significant proportion of the long-term survivors were those with a good PS. Meanwhile, fatal complications (perforation, penetration) were encountered in about 10–20% of unresectable cStage IVA patients.

One of the extracted reports pertained to comparison of radiation alone vs. chemoradiotherapy in patients with unresectable, locally advanced esophageal cancer [105]. This was a somewhat old, low-quality randomized controlled study, and the results showed no significant intergroup difference in the survival time, although it should be noted that the radiation/chemotherapy schedule was greatly different from the currently used schedules. Of the remaining five papers, three pertained to studies of chemoradiotherapy [106–108] and two were reports of single-group prospective studies of chemoradiotherapy administered after induction chemotherapy [109, 110] (Note: the JCOG0303 Study, a randomized controlled study, is treated as a single-group prospective study in this section because both treatment groups in this study received chemoradiotherapy).

The chemotherapy regimens used in the studies referred to herein were mainly fluoropyrimidine + cisplatin, use of which is within the scope of coverage by the national health insurance in Japan.

As for definitive chemoradiotherapy for patients with unresectable cStage IVA esophageal cancer with a good PS, there are no reports of direct comparison with other treatment options (no treatment, radiation alone, or chemotherapy alone), although there is a likelihood of some success in terms of cure or long-term survival with definitive chemoradiotherapy. From the above results and taking into account the risk–benefit balance, strength of evidence, and patient preferences, we conclude that there is only weak evidence to recommend definitive chemoradiotherapy for the treatment of unresectable cStage IVA esophageal cancer. However, the risk of fatal complications, with a reported incidence of about 10–20%, may be inevitable with this treatment modality, which should therefore be selected only after sufficient discussion between the physician and patient about the merits and demerits of the treatment.

Recent advances in chemotherapy have led to the development of a treatment modality with high antitumor efficacy, in which induction chemotherapy is administered to resolve unresectable factors followed by radical surgery in patients with initially unresectable, locally advanced esophageal cancer. A phase II study reported very promising results with a median overall survival of about 34 months and a treatment-related mortality rate of 0% [111], and a randomized controlled study (JCOG1510) [112] comparing definitive chemoradiotherapy and surgery after induction chemotherapy in patients with unresectable, locally advanced esophageal cancer is ongoing.

**CQ13: Is additional chemotherapy recommended for cStage II** **,** **III or IVA esophageal cancer patients who show complete response after definitive chemoradiotherapy?**

### Recommendation statement

There is only weak evidence to recommend additional chemotherapy for cStage II, III, or IVA esophageal carcinoma patients who show complete response to definitive chemoradiotherapy. (*Rate of consensus: 96.4% [27/28]; evidence level: C*).

### Explanatory note

A search of the literature to provide a response to this CQ yielded 36 PubMed articles, 92 Cochrane articles, and 253 ICHUSHI articles. Ten papers were extracted through primary screening, and three papers extracted through secondary screening were subjected to a qualitative systematic review [113–115]. There was no report of any study comparing additional chemotherapy vs. follow-up observation in patients showing complete response to chemoradiotherapy.

All the three papers were reports of retrospective studies from China, and two of the reports compared the therapeutic outcomes between patients who received and did not receive additional chemotherapy after chemoradiotherapy. In one of the reports, patients treated by chemoradiotherapy were divided into two groups according to the therapeutic response (complete response [CR]/partial response [PR] group and stable disease [SD]/progressive disease [PD] group), and therapeutic outcomes were compared between patients who received and did not receive additional chemotherapy [113]. Both of the studies which included patients treated by chemoradiotherapy showed that the patients who received additional chemotherapy showed a better overall survival (OS) [114, 115]. On the other hand, in the report in which the efficacy of additional chemotherapy was evaluated in two groups divided according to the therapeutic response, additional chemotherapy resulted in a better OS in the SD/PD cases, but not in the CR/PR cases. Based on these three reports, the benefit of additional chemotherapy in patients showing complete response to chemoradiotherapy could not be established.

There is no evidence to support additional chemotherapy for patients showing complete response to concurrent chemoradiotherapy, and the significance of such therapy has not been clarified. In past large-scale clinical studies of current chemoradiotherapy, however, two cycles of additional chemotherapy were included and are generally recognized as an international standard [116, 117]. Nevertheless, careful consideration should be given, because the risks may outweigh the benefits depending on the patient’s condition.

Thus, taking into account the risk–benefit balance, strength of evidence, and patient preferences, there is only weak evidence to recommend additional chemotherapy for cStage II, III, or IVA esophageal carcinoma patients who show complete response to definitive chemoradiotherapy.

**CQ14: Is surgical resection recommended for patients with unresectable, locally advanced esophageal cancer (cT4 [e.g., aorta, trachea, bronchus] N0-3M0) that becomes resectable after definitive chemoradiotherapy or induction chemotherapy?**

### Recommendation statement

There is only weak evidence to recommend surgical resection for patients with unresectable, locally advanced esophageal cancer (cT4 [e.g., aorta, trachea, bronchus] N0-3M0) that becomes resectable after definitive chemoradiotherapy or induction chemotherapy. (*Rate of consensus: 89.3% [25/28]; evidence level: C*).

### Explanatory note

A search of the literature to provide a response to this CQ yielded 1527 PubMed articles, 35 Cochrane articles, and 168 ICHUSHI articles, along with one additional article. Sixty-four papers were extracted through primary screening, and 13 papers were extracted through secondary screening (including 9 reports of Japanese single-center retrospective cohort studies, 1 report of an observational study based on the United States National Cancer Data Base, 2 reports of the short-term and long-term outcomes, respectively, in a Japanese multicenter phase II study, and 1 systematic review).

The usefulness of surgery in patients with locally advanced esophageal cancer that is judged to be unresectable at diagnosis, but becomes resectable following definitive chemoradiotherapy or induction chemotherapy was examined; however, as there was no report of any randomized controlled study conducted to compare surgical and non-surgical treatments, reports of the outcomes of surgery after the initial treatment were reviewed.

The current standard treatment for unresectable, locally advanced esophageal cancer in Japan, based on the data of the JCOG0303 Study, is concurrent standard-dose cisplatin + 5FU + 60 Gy radiotherapy; patients who received the protocol-specified treatment showed a 1-year overall survival rate of 55.9% and a 3-years overall survival rate of 25.9%, and 16.9% of the patients (12 of the 71 patients) underwent surgery for residual or recurrent lesions after completion of the protocol-specified treatment [106].

The Japanese retrospective cohort studies reported 3-drug combined regimens consisting of docetaxel in addition to cisplatin and 5-FU as the main drugs, or regimens containing nedaplatin or adriamycin, as the chemotherapy component of chemoradiotherapy for the initial treatment, as well as 3-drug combined regimens, consisting mainly of cisplatin, 5-FU and docetaxel, as induction chemotherapy. The initial treatment regimens as well as the radiation doses varied among the studies. The sample sizes ranged from as small as 12, to 72 [118–127], and a large number of patients dropped out of some of the studies. Patients who underwent surgery after the initial treatment showed a 1-year overall survival rate of 45.7–88.9% [119, 120, 123, 127], a 3-years overall survival rate of 35.2–65.0% [118, 121, 125, 127], a 5-years overall survival rate of 5.7–51.6% [119–122], and an R0 resection rate of 42.4–92.1% [118–126]; thus, the outcomes varied among the studies. The in-hospital mortality rate was 0.0–8.6% [118–125], the overall incidence of ≥ Grade III postoperative complications according to the Clavien-Dindo classification was 20.0–33.0% [120, 122, 123], and the major postoperative complications were pneumonia in 12.5–28.6% of the cases [118–120, 125], anastomotic leakage in 8.3–25.0% of the cases [118–121, 123, 125], and recurrent laryngeal nerve paralysis in 9.7–44.4% of the cases [118–121].

In a Japanese, multicenter, phase II study evaluating the efficacy of induction chemotherapy (three cycles of cisplatin + 5-FU + docetaxel) followed by radical resection (COSMOS) based on evaluation of the effect of induction chemotherapy, resectable patients underwent surgery, while unresectable patients received definitive chemoradiotherapy, and if the cancer became resectable after irradiation at 40–60 Gy, conversion surgery was undertaken. Of the 48 patients enrolled, 42% (20 patients) underwent surgery, corresponding to an R0 resection rate of 95.0% (19/20 cases). In the enrolled patient population overall, the 1- and 3-years overall survival rates were 66.7%, 46.6%, respectively, and the 1- and 3-years progression-free survival rates were 50.6% and 39.6%, respectively. In the 19 patients with R0 resection, the 1- and 3-year overall survival rates were 100.0% and 71.4%, respectively, and the 1- and 3-year progression-free survival rates were 83.6% and 61.3%, respectively. There were no intraoperative complications such as organ injury, and the Grade 3 postoperative complications according to the CTCAE were recurrent laryngeal nerve paralysis, respiratory infection, wound infection, pulmonary fistula, and dysphagia, in 4.8% of the patients each; there were no ≥ Grade 4 serious complications or postoperative in-hospital deaths [111, 128].

The systematic review revealed that patients who underwent surgery following the initial treatment generally showed good long-term outcomes. Although it should be noted that the study included mainly patients who became operable, the entire treatment including the initial treatment was associated with a 3-years overall survival rate of 31.0–46.6%, and the long-term outcomes of all patients, including those who did not undergo surgery, were better than those in the JCOG0303 Study. The in-hospital mortality rate and incidence of complications were somewhat higher than that in patients undergoing general surgical procedures for cStage II or III esophageal cancer, but were still considered to be within acceptable range because of the absence of other effective treatment options and good outcomes after R0 resection. When R0 resection is expected to be achieved based on the results of preoperative examination, there is only weak evidence to recommend surgical resection for patients with unresectable, locally advanced esophageal cancer (cT4 [e.g., aorta, trachea, bronchus] N0-3M0) that becomes resectable after definitive chemoradiotherapy or induction chemotherapy; in addition, it is essential to give careful consideration to the safety of surgery and perioperative management and to obtain the patient’s informed consent after explaining the risks of surgery.

At present, a randomized phase III study (JCOG1510) comparing definitive chemoradiotherapy, followed if necessary, by salvage surgery vs. induction chemotherapy with patients who become resectable undergoing conversion surgery, is ongoing.

## Treatment algorithm for cStage IVB esophageal cancer (Figs. 4 and 5)

**Fig. 4:**
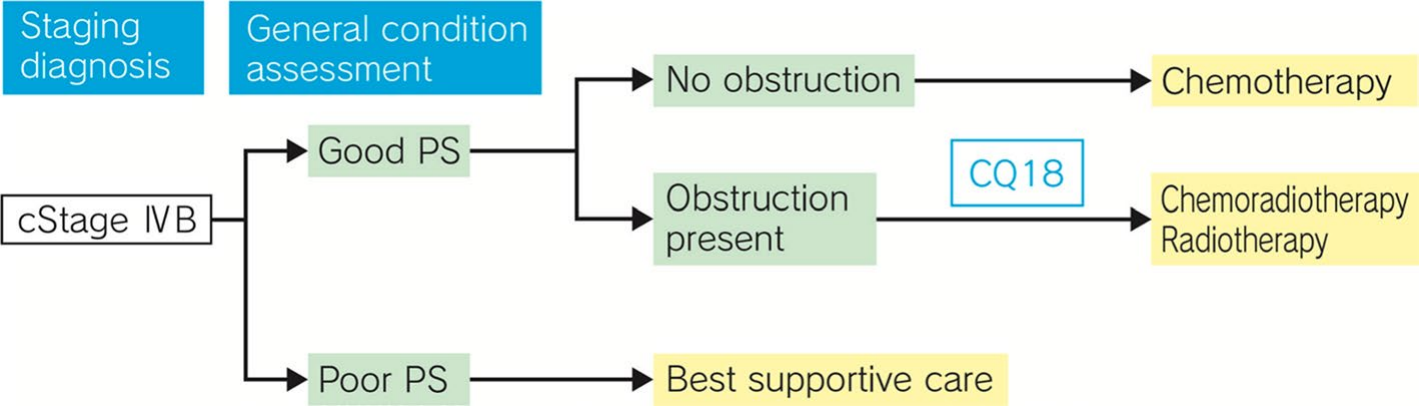
Treatment Algorithm for cStage IVB Esophageal Cancer

**Fig. 5:**
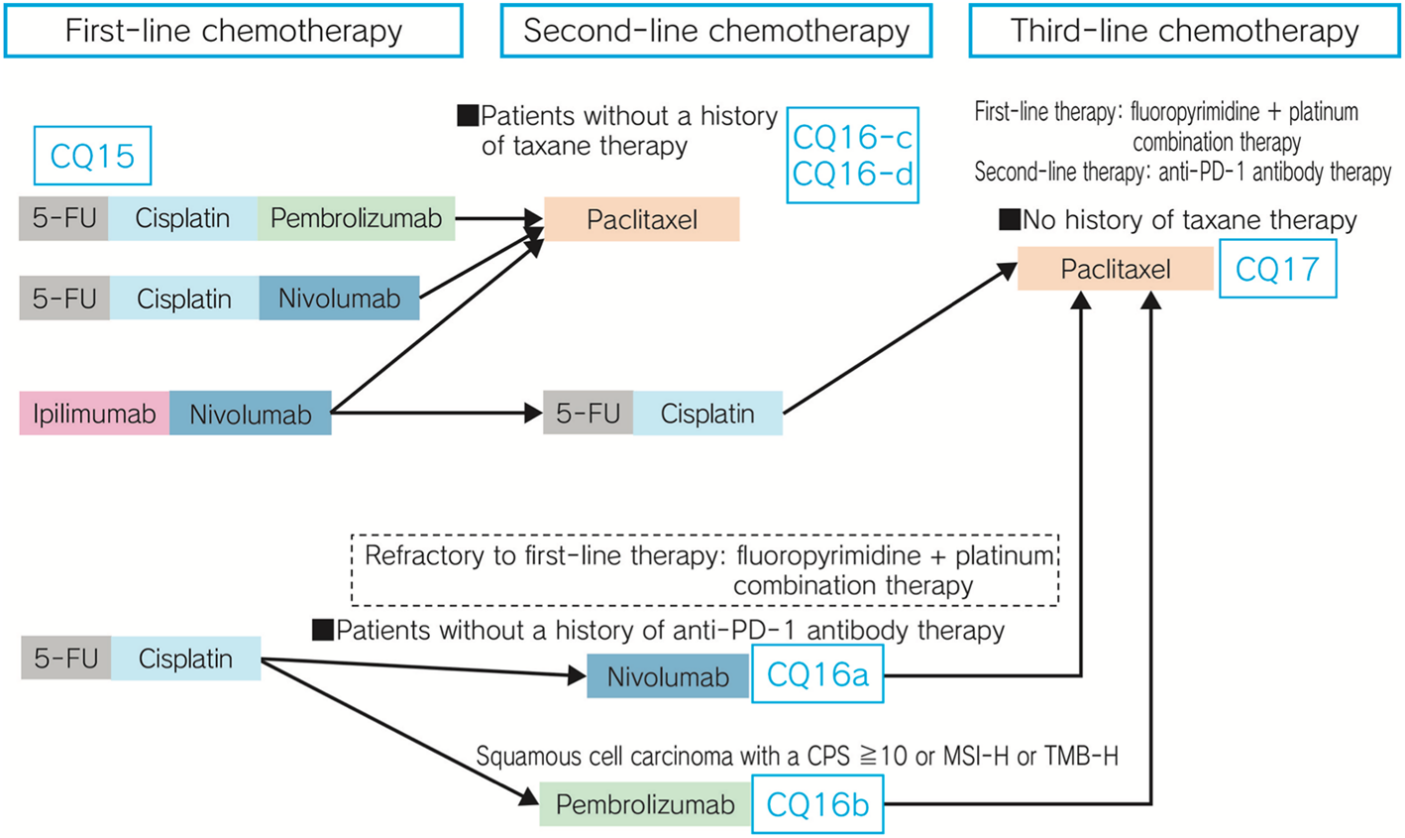
Chemotherapy Regimen for cStage IVB Esophageal Cancer

### Summary

cStage IVB esophageal cancer represents cancer progression to the stage beyond localized disease and requires systemic treatment. First, the patient’s general condition and organ functions should be assessed. If judged to be potentially tolerable, chemotherapy is considered. If the patient has malnutrition due to reduced oral intake, dehydration, or respiratory infection due to aspiration, treatment should be administered to alleviate the symptoms to the extent possible before chemotherapy is considered. Chemotherapeutic drugs and doses should be selected according to the tolerability of the patient judged by the general condition and organ functions. Pain should be actively controlled by reference to the World Health Organization (WHO) guideline for cancer pain relief, etc. Chemotherapy should be considered as a systemic treatment; however, in patients with local lesions such as esophageal stenosis, airway obstruction, or bone metastasis causing pain, which may greatly interfere with the daily life or directly affect life prognosis, chemoradiotherapy is also considered as a local treatment. Patients in poor general condition for chemotherapy may be treated by radiotherapy alone. In this case, treatment to reduce the symptoms associated with cancer should continue to be actively undertaken.

The purpose of treatment was not to completely cure the cancer, but to allow patients to live their daily lives while keeping the cancer manifestations under control, and it is important to monitor the patient’s condition periodically to evaluate and decide about whether treatment should be continued.

**CQ15: What chemotherapy would be recommended as first-line therapy for patients with unresectable, advanced/recurrent esophageal cancer?**

### Recommendation statement

① There is strong evidence to recommend pembrolizumab + cisplatin + 5-FU therapy as first-line therapy for the treatment of patients with unresectable, advanced/recurrent esophageal cancer. (*Rate of consensus: 92.3% [24/26]; strength of evidence: A*).

② There is strong evidence to recommend nivolumab + cisplatin + 5-FU therapy or nivolumab + ipilimumab therapy as first-line therapy for unresectable, advanced/recurrent esophageal cancer, but the patient’s general condition, tumor PD-L1 expression level (tumor proportion score [TPS]), and treatment tolerability should be taken into account. (*Rate of consensus: 88.0% [22/25]; strength of evidence: A*).

### Explanatory note

A search of the literature to provide an answer to this CQ yielded 866 PubMed articles, 193 Cochrane articles, and 156 ICHUSHI articles, along with 3 additional papers. Fifty-eight papers were retrieved through primary screening, and 28 papers were extracted through secondary screening. There were five reports of randomized controlled trials pertinent to this CQ and 23 reports of prospective studies of intervention by chemotherapy. These studies were subjected to a qualitative systematic review.

There are no chemotherapy regimens that have demonstrated clear prolongation of the survival as compared with best supportive care alone in patients with unresectable, advanced/recurrent esophageal cancer. Relatively old studies comparing cisplatin monotherapy and cisplatin + 5-FU combination therapy failed to demonstrate the superiority of combination therapy. Studies comparing cisplatin + 5-FU combination therapy with and without cetuximab as first-line therapy did not show any clear add-on effect of cetuximab. Similarly, studies investigating the additional effect of panitumumab, another anti-EGFR antibody, on cisplatin + 5-FU combination therapy could not confirm efficacy. Combined cisplatin plus 5-FU therapy yielded a response rate of about 30% and a median survival time of 6.6–9.5 months [129–133], which have been recognized as the standard treatment. Chemotherapy regimens using cisplatin in combination with paclitaxel [134], irinotecan [135], or capecitabine [136], instead of 5-FU, have been reported to show comparable efficacy to combined cisplatin + 5-FU therapy; however, these regimens have not been recognized as standards. The adverse events were ≥ Grade 3 neutropenia, nausea, and malaise, in about 10% of the cases each. Combined treatment with 5-FU and nedaplatin, as an alternative to cisplatin, was also assessed in a phase II study, with the results showing a response rate of about 39.5% and a median survival time of 8.8 months, so that nedaplatin has come to be recognized as the drug of choice in patients in whom cisplatin cannot be used due to renal function and/or cardiac function [137]. Clinical studies of combined 5-FU plus oxaliplatin reported from overseas showed that oxaliplatin as well as nedaplatin can be used even in patients with renal disfunction and those who cannot receive large amounts of fluids. However, oxaliplatin must be administered with intermission, dose reduction, or withdrawal because oxaliplatin accumulation causes peripheral neuropathy.

Triplet chemotherapy regimens have recently been reported to show high efficacy, with a reported response rate of about 60% and median survival of ≥ 10 months [138–142]. However, the superior efficacy in terms of overall survival to combined cisplatin + 5-FU therapy, which is the standard treatment, has so far not been established. This regimen should be used with care. At present, a randomized controlled study, JCOG1314, comparing a triplet regimen, in which docetaxel is added every 2 weeks to cisplatin + 5-FU, vs. combined cisplatin + 5-FU therapy is ongoing [143] and the results are still awaited.

Recently, immune checkpoint inhibitors were shown to be useful as second-line treatment, and their usefulness as first-line chemotherapy was also evaluated. The KEYNOTE-590 study compared additional use of pembrolizumab vs. placebo in combination with the standard treatment cisplatin + 5-FU in patients with advanced/recurrent esophageal cancer as the first-line treatment. In squamous cell carcinoma patients with a combined positive score (CPS) of ≥ 10, the median overall survival was 13.9 months (95% CI 11.1–17.7 months) in the pembrolizumab + chemotherapy group and 8.8 months (95% CI 7.8–10.5 months) in the placebo + chemotherapy group, demonstrating the superiority of pembrolizumab + chemotherapy (HR 0.57, 95% CI 0.43–0.75, *p* < 0.0001). In patients with squamous cell carcinoma, in patients with a CPS of ≥ 10, and in the overall population, pembrolizumab + chemotherapy yielded a significantly better overall survival than placebo + chemotherapy. The incidence of adverse events in the pembrolizumab + chemotherapy group was somewhat higher, but it was still considered to be within acceptable range [144]. The CheckMate 648 study compared the treatment outcomes of standard chemotherapy cisplatin + 5-FU, nivolumab + chemotherapy, and 2 immune checkpoint inhibitors (nivolumab + ipilimumab) alone in patients with advanced/recurrent, esophageal squamous cell carcinoma, as the first-line treatment. In patients with a Tumor Proportion Score (TPS) of ≥ 1, the median overall survival time was significantly longer in the nivolumab + chemotherapy group (15.4 months; 95% CI 11.9–19.5 months) and nivolumab + ipilimumab group (13.7 months; 95% CI 11.2–17.0 months) as compared with that in the chemotherapy alone group (9.1 months; 95% CI 7.7–10.0 months). In the all randomized population, the median overall survival time was also significantly longer in the nivolumab + chemotherapy group (13.2 months; 95% CI 11.1–15.7 months) and nivolumab + ipilimumab group (12.7 months; 95% CI 11.3–15.5 months) as compared with that in the chemotherapy alone group (10.7 months; 95% CI 9.4–11.9 months). However, it should be noted that nivolumab + chemotherapy and nivolumab + ipilimumab tended to have variable effects depending on the tumor PD-L1 expression level (TPS). Namely, in patients with a TPS of < 1, the overall survival of nivolumab + chemotherapy and nivolumab + ipilimumab was comparable to that of chemotherapy. The incidence of adverse events tended to be higher in the nivolumab + chemotherapy group as compared with that in the nivolumab + ipilimumab group or chemotherapy alone group, but it was still considered to be within acceptable range [145].

Thus, taking into account the risk–benefit balance, strength of evidence, and patient preferences, we concluded that “there is strong evidence to recommend pembrolizumab + cisplatin + 5-FU therapy as first-line therapy for the treatment of unresectable, advanced/recurrent esophageal cancer” and that “there is strong evidence to recommend nivolumab + cisplatin + 5-FU therapy or nivolumab + ipilimumab therapy as first-line therapy for the treatment of unresectable, advanced/recurrent esophageal cancer, while it is also necessary to take into account the patient’s general condition, tumor PD-L1 expression level (TPS), and patient’s treatment tolerability”.

**CQ16: What chemotherapy would be recommended as second-line therapy for patients with unresectable, advanced/recurrent esophageal cancer refractory to first-line chemotherapy, including fluoropyrimidine + platinum therapy?**

### Recommendation statement

(In patients without a history of anti-PD-1 antibody therapy).

- In patients without a history of anti-PD-1 antibody therapy, there is strong evidence to recommend nivolumab therapy (squamous cell carcinoma). (*Rate of consensus: 100% [25/25]; strength of evidence: A*)
- In patients without a history of anti-PD-1 antibody therapy, there is only weak evidence to recommend pembrolizumab therapy (squamous cell carcinoma with a CPS of ≥ 10 or MSI-H or TMB-H). (*Rate of consensus: 96.2% [25/26]; strength of evidence: B*)

(In patients without a history of taxane therapy).

c. In patients without a history of anti-PD-1 antibody therapy or taxane therapy, there is only weak evidence to recommend paclitaxel therapy. (*Rate of consensus: 96.4% [27/28]; strength of evidence: C*)
d. In patients with a history of anti-PD-1 antibody therapy, but no history of taxane therapy, there is only weak evidence to recommend paclitaxel therapy. (*Rate of consensus: 100% [28/28]; strength of evidence: C*)

### Explanatory note

A search of the literature to provide a response to this CQ yielded 248 PubMed articles, 122 Cochrane articles, and 126 ICHUSHI articles, along with 2 other additional papers, all of which were subjected to primary screening. Four papers dealing with randomized controlled studies and 11 papers dealing with intervention studies were retrieved through a secondary screening and subjected to a qualitative systematic review. The following two randomized controlled studies demonstrated the efficacy of the anti-PD-1 antibodies nivolumab and pembrolizumab, respectively, as second-line therapy in patients with unresectable, advanced/recurrent esophageal cancer refractory to first-line chemotherapy, including combined fluoropyrimidine + platinum therapy [146, 147].

The ATTRACTION-3 study demonstrated the superiority of nivolumab (240 mg/body, every 2 weeks) over chemotherapy with paclitaxel or docetaxel in terms of the overall survival, which was the primary endpoint of the study, in patients with squamous cell carcinoma (median overall survival: 10.9 months in the nivolumab group and 8.4 months in the chemotherapy group; HR 0.77 [95% CI 0.62–0.96]) [146]. Although nivolumab did not show the superior progression-free survival, about a half of the patients received taxanes as after-treatment. This suggested the efficacy of the subsequent-treatment for the overall survival. The incidence of ≥ Grade 3 treatment-related adverse events was 18% in the nivolumab group and 64% in the chemotherapy group. Although nivolumab was superior in the overall population, no difference was observed in the progression-free survival and nivolumab was suggested to be inferior in some patients, and administration of paclitaxel may be considered in such patients.

The KEYNOTE-181 study in patients with squamous cell carcinoma or adenocarcinoma failed to demonstrate the statistical superiority of pembrolizumab (200 mg/body, every 3 weeks) over chemotherapy with paclitaxel, docetaxel, or irinotecan in terms of the overall survival in the following three prespecified main analysis populations: (i) patients with positive tumor PD-L1 expression (CPS ≥ 10); (ii) patients with squamous cell carcinoma; and (iii) intention-to-treat population [147]. Although the analysis was exploratory, pembrolizumab showed a longer overall survival than chemotherapy (HR 0.64 [95% CI 0.46–0.90]) (median overall survival: 10.3 months in the pembrolizumab group and 6.7 months in the chemotherapy group) in squamous cell carcinoma patients with positive tumor PD-L1 expression. The incidence of ≥ Grade 3 adverse events whose relationship to the treatment could not be ruled out was 18% in the pembrolizumab group and 41% in the chemotherapy group. The phase II KEYNOTE-158 study in patients with solid tumors refractory to previous treatment showed a promising reduction of the tumor size in patients with microsatellite instability high (MSI-H) or tumor gene mutation burden high (TMB-H) treated with pembrolizumab (200 mg/body, every 3 weeks), demonstrating the efficacy of this drug, regardless of the cancer type [148, 149].

According to a randomized controlled study of gefitinib, which is an inhibitor of the epidermal growth factor receptor (EGFR) pathway, gefitinib did not prolong the overall survival as compared with placebo in patients with adenocarcinoma or squamous cell carcinoma of the esophagus or esophagogastric junction with a history of previous treatment (median overall survival: 3.73 months in the gefitinib group and 3.67 months in the placebo group; HR 0.90 [95% CI 0.47–1.09]) [150].

Of the 11 reports of intervention studies, two reports pertained to single-arm phase II studies of paclitaxel and docetaxel, respectively [151, 152], and 1 report pertained to a randomized phase II study of paclitaxel (100 mg/m^2^ administered weekly for 6 weeks in a 7-week cycle) and docetaxel (70 mg/m^2^, every 3 weeks) as second-line chemotherapy in patients with esophageal squamous cell carcinoma refractory to fluoropyrimidine- and platinum-based chemotherapy [8, 153]. According to this randomized phase II study, overall survival (median: 8.8 months in the paclitaxel group and 7.3 months in the docetaxel group; HR 0.62 [95% CI 0.38–0.99]) and progression-free survival (median 4.4 months in the paclitaxel group and 2.1 months in the docetaxel group; HR 0.49 [95% CI 0.30–0.78]) were significantly longer in the paclitaxel group than in the docetaxel group. Grade 3 or higher neutropenia (28% in the paclitaxel group and 80% in the docetaxel group) and febrile neutropenia (0% in the paclitaxel group and 46% in the docetaxel group) were more common in the docetaxel group.

In addition, there were also reports of combined irinotecan plus docetaxel therapy [154] and combined docetaxel plus platinum drug therapy [155, 156]; neither reported a great difference in efficacy between the combined therapy and monotherapy, however, combined therapy tends to show higher toxicity than monotherapy.

In patients who received anti-PD-1 antibody therapy + chemotherapy as first-line therapy, patients who received anti-PD-1 antibody therapy as adjuvant therapy following esophagectomy, and patients who received chemotherapy including taxanes as preoperative chemotherapy, it is necessary to select the appropriate second-line therapy for recurrence, considering the history of treatment with these drugs and the time to recurrence.

Thus, taking into account the risk–benefit balance and strength of evidence, we formulated different recommendation statements depending on the history of anti-PD-1 antibody therapy and taxane therapy, as follows: in patients without a history of anti-PD-1 antibody therapy, there is strong evidence to recommend nivolumab therapy (squamous cell carcinoma) and there is only weak evidence to recommend pembrolizumab therapy (squamous cell carcinoma with a CPS of ≥ 10 or MSI-H or TMB-H), and in patients with/without a history of anti-PD-1 antibody therapy and without a history of taxane therapy, there is only weak evidence to recommend paclitaxel therapy.

**CQ17: What would be recommended as third-line therapy for patients with unresectable, advanced/recurrent esophageal cancer who received fluoropyrimidine + platinum combination therapy as first-line therapy and anti-PD-1 antibody therapy as second-line therapy?**

### Recommendation statement

In patients without a history of taxane therapy, there is only weak evidence to recommend paclitaxel therapy. (*Rate of consensus: 100% [28/28], strength of evidence: C*).

### Explanatory note

A search of the literature conducted to respond to this CQ yielded 173 PubMed articles, 220 Cochrane articles, and 144 ICHUSHI articles, along with 4 other additional papers, which were subjected to primary and secondary screenings. Finally, one report of a randomized controlled study and five reports of intervention studies were retrieved and subjected to a qualitative systematic review.

Although the results of intervention studies of EGFR inhibitors/monotherapies/combination chemotherapies have been reported, there is no clear evidence to indicate whether third-line therapy might be effective for prolonging the survival [150, 157–161]. In addition, the safety and efficacy of anti-PD-1 antibody therapy have not yet been reported. However, in the ATTRACTION-3 study, the study treatment group received the anti-PD-1 antibody nivolumab as second-line therapy after combined fluoropyrimidine + platinum therapy, and about a half of the subjects received taxanes as after-treatment [146]. Although there was no significant difference in the progression-free survival between the nivolumab and chemotherapy groups, the overall survival was significantly prolonged in the nivolumab group. The results indirectly suggest that taxane therapy following anti-PD-1 antibody therapy is safe and prolongs the survival.

As for taxanes as second-line chemotherapy, there is a report of a randomized phase II study of paclitaxel and docetaxel in patients who were refractory to combined fluoropyrimidine + platinum therapy [153]. The overall survival and progression-free survival were significantly longer in the paclitaxel group than in the docetaxel group, and the incidences of ≥ Grade 3 neutropenia and febrile neutropenia were lower in the paclitaxel group. Although taxanes were used as second-line chemotherapy in the study, the results suggest that paclitaxel is a more appropriate choice than docetaxel even when taxanes are used as third-line chemotherapy.

Thus, taking into account the risk–benefit balance and strength of evidence, we concluded that in patients without a history of taxane therapy, there is only weak evidence to recommend paclitaxel therapy.

**CQ18 Is palliative radiotherapy recommended for the treatment of patients with cStage IVB esophageal cancer presenting with obstruction?**

### Recommendation statement

There is only weak evidence to recommend palliative radiotherapy for the treatment of patients with cStage IVB esophageal cancer presenting with obstruction. (*Rate of consensus: 100% [28/28]; strength of evidence: C*).

### Explanatory note

A search of the literature conducted to respond to this CQ yielded 192 PubMed articles, 139 Cochrane articles, and 236 ICHUSHI articles; of these, 25 papers were extracted through primary screening, and 5 of these were extracted through secondary screening and subjected to a qualitative systematic review.

A randomized comparative study of radiotherapy alone vs. chemoradiotherapy for the treatment of esophageal cancer that could not be treated radically in patients presenting with obstruction (TROG 03.01) revealed no significant difference in the percentage of patients who showed improvement in the dysphagia between the radiotherapy alone and chemoradiotherapy groups (35% vs. 45%, *p* = 0.13) and a significantly lower incidence of Grade 3–4 adverse events in the radiotherapy alone group (16% vs. 36%, *p* = 0.0017) [162, 163]. There was no difference in the median survival time between the radiotherapy alone group (6.7 months) and chemoradiotherapy group (6.9 months). Although 56 of the 220 subjects (26%) in this study had squamous cell carcinoma, 156 subjects (71%) had cStage IVB disease, and radiotherapy with external irradiation at 35 Gy/15 Fr or 30 Gy/10 Fr and combined chemoradiotherapy with cisplatin + 5-FU were adopted; therefore, this study was closely related to this CQ.

There were two reports of randomized comparative studies conducted to compare the therapeutic responses to radiotherapy and stenting [2, 3, 164, 165]. Earlier improvement of dysphagia was observed in the metallic stent group in both reports, but the improvement in the symptoms was better sustained in the radiotherapy (intracavitary brachytherapy) group. The contents of these studies were perhaps not very pertinent to this CQ, because intracavitary brachytherapy, which was used in both the studies, is scarcely adopted in Japan; however, the results suggest that the local antitumor effect of radiotherapy is more effective for providing sustained relief from dysphagia as compared with that of stenting.

In regard to the irradiation methods, according to a prospective cohort study of intracavitary brachytherapy and external irradiation, the percentage of patients who showed improvement of the dysphagia tended to be higher in the external irradiation group than in the intracavitary brachytherapy group (83% vs. 64%, *p* = 0.048), and the incidence of ≥ Grade 3 adverse events was lower in the external irradiation group (3% vs. 13%) [4, 166].

To sum up, radiotherapy is effective for improvement of dysphagia, and serious adverse reactions are not necessarily common, although some adverse events do occur. Desire for symptomatic amelioration is generally profound in patients with dysphagia and this treatment is covered by the national health insurance. Taking into account the risk–benefit balance, strength of evidence, cost burden, and patient preferences, we concluded that there is only weak evidence to recommend palliative radiotherapy for the treatment of patients with cStage IVB esophageal cancer presenting with obstruction.
